# Molecular epidemiology of fluoroquinolone resistant *Salmonella* in Africa: A systematic review and meta-analysis

**DOI:** 10.1371/journal.pone.0192575

**Published:** 2018-02-12

**Authors:** Getachew Tadesse, Tesfaye S. Tessema, Getenet Beyene, Abraham Aseffa

**Affiliations:** 1 Department of Biomedical Sciences, College of Veterinary Medicine and Agriculture, Addis Ababa University, Debre Zeit, Ethiopia; 2 Institute of Biotechnology, Addis Ababa University, Addis Ababa, Ethiopia; 3 Department of Medical Laboratory Sciences, Faculty of Health Sciences, Jimma University, Jimma, Ethiopia; 4 Armauer Hansen Research Institute (AHRI), ALERT Campus, Addis Ababa, Ethiopia; Universidad de la Republica Uruguay, URUGUAY

## Abstract

**Background:**

Wide-ranging evidence on the occurrence of fluoroquinolone (FQ) resistance genetic determinants in African *Salmonella* strains is not available. The main objectives of this study were to assess the heterogeneity, estimate pooled proportions and describe the preponderance of FQ-resistance determinants in typhoidal and non-typhoidal *Salmonella* (NTS) isolates of Africa.

**Methods:**

Genetic and phenotypic data on 6103 *Salmonella* isolates were considered. Meta- and frequency analyses were performed depending on the number of studies by category, number of isolates and risks of bias. A random effects model was used to assess heterogeneity and estimate pooled proportions. Relative and cumulative frequencies were calculated to describe the overall preponderance of FQ-resistance determinants in quinolone resistant isolates.

**Results:**

The pooled proportion of *gyrA* mutants (*Salmonella enterica* serovar Typhi, *Salmonella enterica* serovar Typhimurium, and *Salmonella enterica* serovar Enteritidis) was estimated at 5.7% (95% Confidence interval (CI) = 2.6, 9.8; Tau squared (T^2^) = 0.1105), and was higher in *S*. Typhi than in *S*. Typhimurium (odds ratio (OR) = 3.3, 95%CI = 2, 5.7). The proportions of each of *gyrB* and *parC* mutants, and strains with Plasmid Mediated Quinolone Resistance genes (*qnrA*, *qnrB* and *qnrS*) were low (≤ 0.3%). Overall, 23 mutant serotypes were identified, and most strains had mutations at codons encoding Ser83 and Asp87 of *gyrA* (82%, 95%CI = 78, 86).

**Conclusions:**

Mutations at *gyrA* appear to account for ciprofloxacin non-susceptibility in most clinical *Salmonella* strains in Africa. The estimates could be harnessed to develop a mismatch-amplification mutation-assay for the detection of FQ-resistant strains in Africa.

## Introduction

*Salmonella* is one of the major causes of morbidity and mortality in Africa [1]. The serotypes the most frequently associated with invasive disease are *S*. Typhi, *S*. Typhimurium and *S*. Enteritidis [1, 2]. Incidence of invasive non-typhoidal *Salmonella* (iNTS) disease as high as 227 per 100,000 [range 152–341] [3] and a case fatality of 19% (276/1427) among low risk populations [4], higher *S*. *Typ*hi blood stream infections in children than in adults (OR, 1.7; 95% CI = 1,2.7) [5], higher iNTS disease in high than in low malaria endemic settings (OR = 4.9 (1.6,14.9) [6], higher malaria-NTS co-infection in children than in adults [7] and a wide-spread occurrence of antimicrobial resistant strains [8,9] have been documented.

Increased resistance to chloramphenicol led to the increased use of ampicillin and cotrimoxazole [10], and the subsequent emergence of strains multiply resistant to these drugs led to the use of FQs [11]. However, FQ-resistance ensued shortly thereafter [11], and this has been linked to chromosomal mutations and plasmid-borne genes [12]. Infections with strains with high minimum inhibitory concentrations (MICs) have been associated with more treatment failures than those due to strains with low MICs (relative risk = 5.75; 95% CI = 1.8, to 18.7) [13]. Due to multiple mechanisms, recognition of FQ-resistance in *Salmonella* is complicated [14], and treatment failures have necessitated modifications of the break point levels of susceptibility/resistance [15, 16].

Selection pressure triggered by drugs, geographic and social transmission environments have been proposed as factors attributable to variation of *gyrA* mutations in *Mycobacterium tuberculosis* [17]. Correspondingly, the variations in the occurrence of FQ-resistant *Salmonella* among WHO regions [18] and within Africa [8,19] could be due to distinctions in the occurrence of genetic determinants across serotypes and differences in selection pressure across locations. However, wide-ranging comparative studies (typhoidal vs. NTS) or systematic reviews/meta-analyses on the occurrence of FQ-resistance genetic determinants across serotypes, and countries/regions in Africa are not available. The main objectives of this study were to assess the heterogeneity, estimate pooled proportions and describe the preponderance of genetic determinants that confer resistance to FQs in typhoidal and non-typhoidal *Salmonella* isolates of Africa.

## Methods

The study was carried out according to the guidelines set-forth in Preferred Reporting Items for Systematic Reviews and Meta-Analyses (PRISMA) [20, 21] (S1 Checklist). The questions were on the heterogeneity and preponderance of genetic determinants of FQ-resistance in typhoidal and non-typhoidal *Salmonella* isolates of Africa. The study populations were salmonellae isolated from humans, animals and animal products. The study designs included prospective and retrospective studies, and case reports. Meta- and/or frequency analyses were performed by sampling populations and methodological features of the studies. The primary outcome of interest was the proportions of FQ-resistance genetic determinants. The secondary outcomes were the phenotypic proportions: multi-drug resistance (MDR), nalidixic acid non-susceptibility (Nal^ns^), ciprofloxacin non-susceptibility (Cip^ns^), MDR + Cip^ns^, and non-MDR + Cip^ns^). MDR was defined as resistance to chloramphenicol, ampicillin and cotrimoxazole. Nalidixic acid susceptibility was defined as minimum inhibitory concentration (MIC) ≤ 16μg/mL [22]. Ciprofloxacin susceptibility was defined as an MIC ≤ 0.06μg/mL [16, 22]. Intermediate susceptibility and resistance were combined as non-susceptible.

### Study search

We searched published studies in PubMed, Google Scholar and African Journals Online (AJOL). A systematic search was done to avoid duplication of the subject: *Salmonella* AND Africa AND (‘systematic review’ OR ‘meta-analysis’). Multiple search strings (medical subject headings [MeSH] and text words [tiab]) were tried to identify primary studies. The search string that enabled to locate most studies was the following: *Salmonella* AND (quinolone OR fluoroquinolone) AND (Africa OR Algeria OR Angola OR Benin OR Botswana OR ‘Burkina Faso’ OR Burundi OR Cameroon OR ‘Cape Verde’ OR ‘Central African Republic’ OR Chad OR Comoros OR ‘Congo Republic’ OR ‘Côte d'Ivoire’ OR ‘Ivory Coast’ OR ‘Democratic Republic of the Congo’ OR Djibouti OR Egypt OR ‘Equatorial Guinea’ OR Eritrea OR Ethiopia OR Gabon OR Gambia OR Ghana OR Guinea OR ‘Guinea Bissau’ OR Kenya OR Lesotho OR Liberia OR Libya OR Madagascar OR Malawi OR Mali OR Mauritius OR Mauritania OR Morocco OR Mozambique OR Namibia OR Niger OR Nigeria OR ‘North Sudan’ OR Rwanda OR ‘Republic of Sudan’ OR ‘Sāo Tome and Principe’ OR Senegal OR Seychelles OR ‘Sierra Leone’ OR Somalia OR ‘South Africa’ OR ‘South Sudan’ OR Sudan OR Swaziland OR Tanzania OR Togo OR Tunisia OR Uganda OR ‘United Republic of Tanzania’ OR ‘Western Sahara’ OR ‘Saharawi Arab Democratic Republic’ OR Zambia OR Zimbabwe). Reference lists were scanned to make-out additional reports. The last search was done on January 21, 2018.

### Inclusion and exclusion criteria

We excluded reviews, and studies with titles and/or abstracts unrelated to the primary outcomes of interest. Studies with the following characteristics were screened for eligibility: (i) published in English or French, (ii) reported genetic determinants of resistance to FQs, (iii) *Salmonella* isolated from humans, or domestic animals or animal products, and (iv) serotyped. Letters, correspondence and reports with unclear data were not included. An outbreak was defined as the occurrence of two or more cases of salmonellosis due to consumption of contaminated food/water from the same source [23]. Salmonellae isolated from samples collected from diverse locations, over a long period of time, and with different AMR and/or genomic patterns were considered as non-outbreak isolates.

### Data extraction

The following data were considered and extracted: (i) study identifier: first author, year of publication, year of study, country, target population (humans, domestic animals and animal products), and sampling population (health-care/nosocomial, community, travel, animal species/products); (ii) methods: sample size, sampling method, sample, drug susceptibility test (DST), quinolones, break point level/interpretive standard, and gene detection (phenotype/genotype based); (iii) results: numbers of isolates (overall, typhoidal and NTS), numbers of isolates subjected to DST, numbers of MDR strains, numbers of nalidixic acid non-susceptible strains (Nal^ns^), numbers of ciprofloxacin non-susceptible strains (Cip^ns^), numbers of MDR + Cip^ns^ strains, non-MDR + Cip^ns^, minimum inhibitory concentrations (MICs)/zone diameters (ZD), number of strains examined to detect mutations (*gyrA*, *gyrB*, *parC* and *parE*), numbers of mutants, mutation positions, substituted amino acids, number of strains examined to detect Plasmid Mediated Quinolone Resistance genes (PMQR) (*qnrA*, *qnrB*, *qnrC*, *qnrD*, *qnrS*, *aac(6’)-Ib-cr*, *qepA*, *oqxA/B*), and numbers of strains with PMQR genes.

Study-specific extraction processes (calculations, exclusions and assumptions) are provided in the table of characteristics of studies (S1 Table). Briefly, data on isolates other than human and domestic animal/animal product origins or collected before 2000 or duplicates were excluded. Authors were contacted to solicit online/supplementary information (S1 Table). Data extraction was done twice–overall and by subgroup (typhoidal and NTS). A further extraction was done by serotype. Multiple cross checks were performed to ensure extraction harmony and fix incompatibilities. The data was extracted by GT.

### Data preprocessing

We categorized the data by region–Central, Northern, Eastern, Western and Southern Africa. Multi-country data were handled by country. Homogeneity based frequencies (group/serotype, country/region) were calculated to circumvent problems associated with study precision [24, 25]. Unreported sample size (Ns) is reported as not reported (nr), but approximated: Ns = n/p; where n = number of isolates, and p = pooled proportion. If MICs were provided, the number of non-susceptible strains classified as susceptible–due to high break point levels of the earlier standards–was adjusted according to the modified break point level [16]. In the absence of phenotype data, numbers of MDR and quinolone non-susceptible strains were extrapolated from genotype data (MDR = *cat* + *bla*_TEM_ + *dfrA-sul*1, and *qnrS*/*gyrA* mutation positive = quinolone non-susceptible). To normalize data distribution, study level estimates were double arcsine (t) transformed, t = sin^-1^(√n/N+1) + sin^-1^(√n+1/N+1), Se (t) = √1/N+0.5, [26].

### Assessment of quality and risk of bias

Study validity was established by the inclusion criteria [27]. The methodological quality of each study was assessed following the Joanna Briggs Institute (JBI) critical appraisal tool [28], and components [21] for which there is a practical evidence of bias: (i) target population (humans vs. animals/animal products), (ii) sampling population (humans [27]–health care vs. non-health care–community, adoptees, travelers; animals/animal products–species, farms, slaughterhouse, markets), (iii) sample size (n ≤ 384 vs. higher; n = Z^2^PQ/d^2^; where P_exp_ = 0.5), (iv) study design (prospective, retrospective, collections, cases), sampling method (consecutive/probability vs. non-probability), (iv) samples (blood vs. others), (v) phenotype detection (E-test/dilution (MIC) vs. disk diffusion (ZD)) [15,16]; ciprofloxacin susceptibility break point level/interpretive standard (Cip^s^ ≤ 0. 06μg/mL vs. higher) [15,16], and (vi) genotype detection (PCR based vs. Whole Genome Sequence based). In each sampling population, studies with the following characteristics were considered as low risk studies: sample size (> 384), prospective/consecutive sampling, and Cip^s^ ≤ 0.06μg/mL. The Begg and Mazmudar rank correlation test was used to assess bias across studies (small study effects).

### Data analysis

Microsoft Office Excel 2007, Stata (Version 11.1, Stata Corp, College Station, Texas), and Epi Info^TM^ (version 3.5.1, Center for Disease Control, CDC, USA) were used in data analysis.

### Estimation of heterogeneity

We measured inconsistency using Cochran’s test (Q), tau squared (T^2^) and Higgin’s indexes (H^2^ and I^2^) [29]. The 95% confidence bounds of I^2^ were calculated using the following formula: ln (H) +/-1.965 (Se (ln H)) [29]. If Q > k, Se was calculated by the following formula: Se (ln H) = 0.5 [(ln Q—ln k-1) / (√2Q-√2k-3)]. If Q < K, Se was calculated by the following formula: Se (ln H) = √(1/2k – 4) [(3(k *−* 2)2–1) / (3(k *−* 2)^2^)], [29]. I^2^ estimates less than 25% and greater than 75%, respectively, were considered as substantially low and high heterogeneity. The significance of a difference in subgroups’ estimates was examined using a one tailed X^2^ test [30]; X^2^ = P(X > x), where X is X^2^ random variable.

### Estimation of pooled proportion

We used a random effects model to pool double arcsine estimates. We performed single study omitted influence analyses to examine the sensitivities of pooled estimates. A pooled estimate was considered as robust, if all single study omitted pooled estimates and 95% CIs lie within or closer to its 95% CIs. Pooled estimates were back transformed to proportions (p): p = 0.5 {1-sgn (cos t) [1- (N (sin t)^2^ + (sin t)2–1/ (N sin t))^2^]^0.5^}; where N = sample size [25]. The Yates corrected Chi square test was used to evaluate the significance of a difference between estimates. Alpha (α) was set at 0.05. Odds ratios (OR) were calculated to evaluate the magnitude of superiority or inferiority of estimates.

### Proportion of *gyrA* mutant infection

The proportion of patients infected with *gyrA* mutants was derived from the pooled proportions of mutant strains, and the pooled proportion of patients with invasive salmonellosis. The proportions of typhoidal and iNTS disease were estimated using data of studies that isolated both typhoidal and non-typhoidal *Salmonella*. The isolates have been recovered from 47337 samples collected between 2007 and 2015 in nine countries (S1 Table).

### Relative and cumulative frequencies

Relative and cumulative frequencies were calculated to describe the overall preponderance of mutated genes, PMQR genes, mutated positions, and substituted amino acids in quinolone resistant salmonellae. The proportion of a mutated or PMQR gene was calculated by dividing the number of strains in which the mutated or PMQR gene was found by the number of quinolone resistant strains examined to detect the genotype. The preponderance of a mutation position (‘hot spot’) was calculated by dividing the frequency of the mutation by the cumulative frequency of mutations within the QRDR. The occurrence of a specific mutation (position and amino acid) was calculated by dividing the frequency of the mutation (position and amino acid) by the cumulative frequency of mutations in the QRDR. The predominance of an amino acid substituted at a specific mutation position was calculated by dividing the frequency of the amino acid by the cumulative frequency of mutations at the specific position.

## Results

### Search and selection of studies

**Fig 1:**
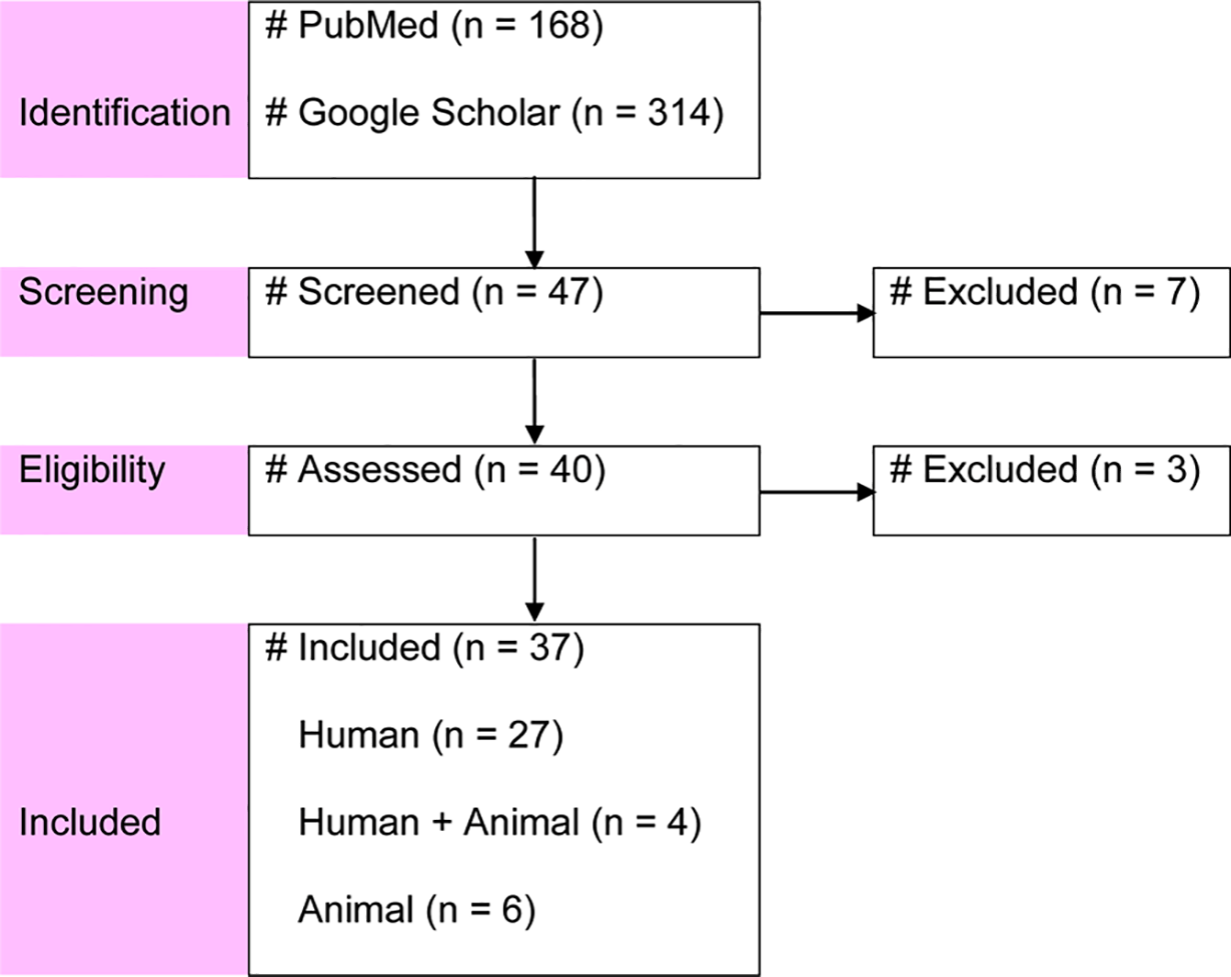
Flow diagram of search and selection of studies.

Fig 1 presents a flow diagram of the search and selection of studies. One systematic review on FQ-resistant enteric bacteria–with 10 reports on *Salmonella* [8]–was identified, but the heterogeneity and predominance of the resistance determinants have not been described. Other systematic reviews/meta-analyses that reported heterogeneity estimates, and genotypic and phenotypic proportions were not found. In total, 47 reports were screened, and 37 were included [31–67].

### Characteristics of included studies

The characteristics of included studies are given in S1 Table. The studies were prospective and retrospective and included surveillance–case series [31–38, 50, 63], laboratory-based [42, 51,59]–and cross-sectional [40,52,58,62,66] studies, case reports [48,49,53,64], studies on hospital and laboratory collections [41,43,46,47,65, 67], multi-country collections [39,44,45,54–56], cross-sectional and/or country-based collections [57, 60, 61]. All human isolates but some from adopted children were recovered from patients who sought medical attention for reasons of fever and gastroenteritis or other systemic infections. One-hundred and thirty-eight isolates were isolated from symptomatic or asymptomatic adopted children [44–46, 50, 65]. Other isolates were from patients hospitalized due to travel-related infections [54–56, 65] and cases [48, 49, 53, 64]. Patient characteristics that included pre-sampling antimicrobial use, infection onset and comorbidities have not been reported in most studies. Of the isolates of animal/animal product origins, nine were from diarrheic calves [58] and others were recovered from a variety of samples and animal species–cattle, swine and poultry (S2 Table).

Overall, data on 6103 isolates recovered from humans (n = 5137) [31–56, 63–67], and animals/animal products (n = 966) [40, 52, 55–62] were considered. The salmonellae have been isolated from more than 100,000 samples. The numbers of typhoidal and NTS isolates, respectively, were 2003 and 4100. Most human isolates of community setting origins were from SSA (99.2%, 5040/5082)–Western (n = 1983), Central (n = 1673), Southern (n = 788) and Eastern Africa (n = 596). Most isolates of animal/animal product origins (59.3%, 573/966) were from Northern Africa. Nalidixic acid and/or ciprofloxacin were the test drugs, except in three studies that as well tested isolates’ susceptibility to norfloxacin [58], ofloxacin [59, 66] and enrofloxacin [66]. Resistance gene detection was phenotype–PCR/sequence based [31–37, 40–42, 44–47, 49–66] and whole genome sequence (WGS) based [38, 39, 43, 48, 67].

### Quality assessment and risk appraisal

**Fig 2:**
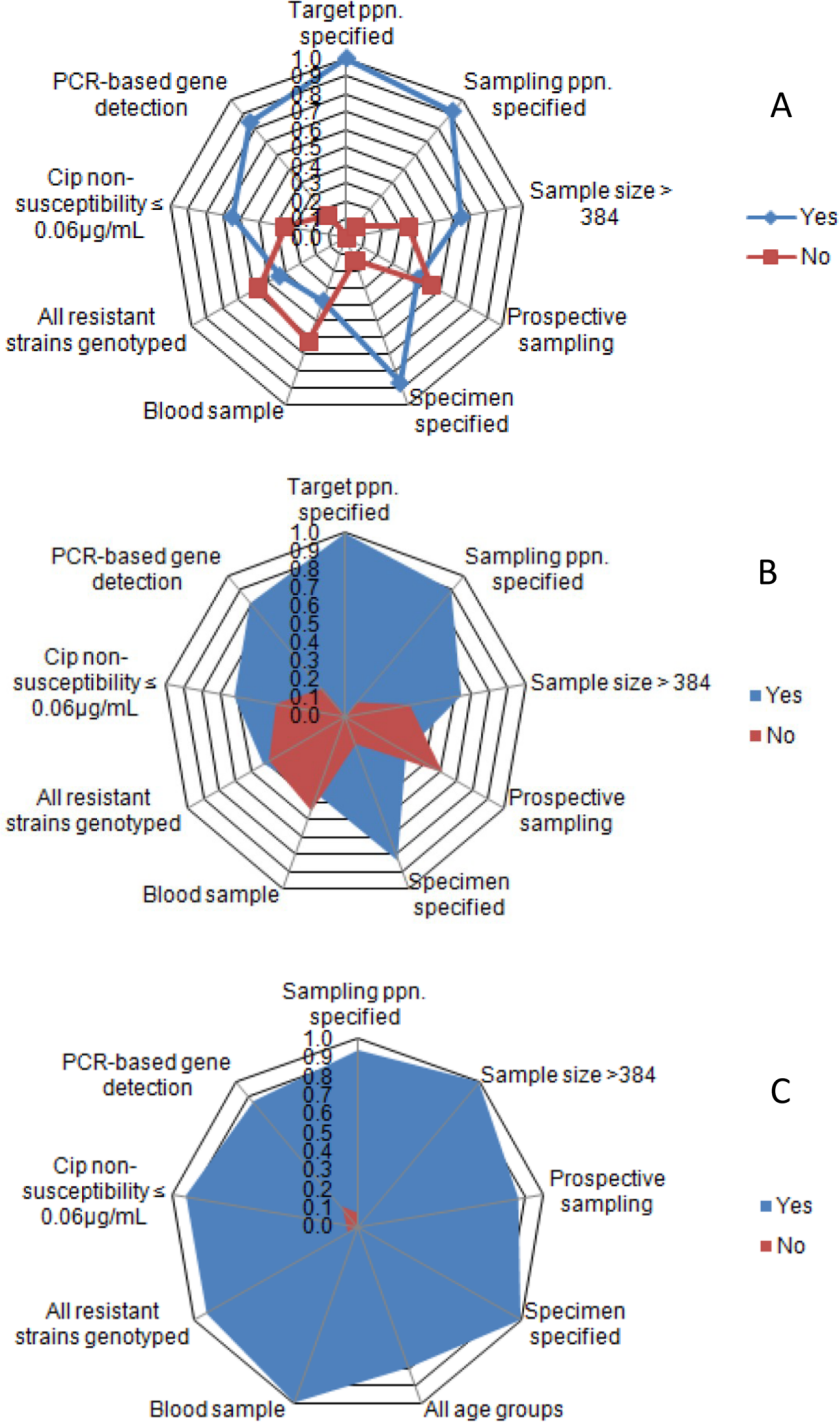
Radar plots of proportions of studies by methods. All studies (A), All human studies (B), Studies used in meta-analysis (C).

The methodological qualities and risks of bias of included studies are given in S2 Table and S3 Table. Fig 2 presents the proportion of studies by methods. Sampling population, sample size (n > 384), sample type, prospective/consecutive sampling, and Cip^s^ cutoff level at ≤ 0.06μg/mL were each reported in 45.9% or more of the studies. Of the studies on isolates of human origins, 25.8% were relatively homogeneous with respect to the sampling population and methods (community, sample size > 384, prospective sampling, blood sample, Cip^s^ cutoff level at ≤ 0.06μg/mL, and gene detection), [31–38]. The studies on animals and animal products were few and generally heterogeneous. Accordingly, meta- and frequency analyses were performed depending on the number of studies by category, number of isolates, and risks of bias/heterogeneity.

Meta-analyses were performed to assess the heterogeneity and estimate pooled phenotypic and genotypic proportions of serotypes commonly associated with invasive disease in humans (*S*. Typhi, *S*. Typhimurium, and *S*. Enteritidis). For this purpose, we used data of studies that employed relatively uniform methods [31–39, 63] (S4 Table). Established standards to assess PCR associated bias [68], and to infer drug susceptibility on genotype based tests [69] are not available. However, the correlation between phenotype and genotype is strongly positive [70], and the margin of equivalence between phenotype and genotype based tests was considered insignificant. The minimum sample size and number of isolates included in the meta-analyses were 626 and 10, respectively, and as all isolates were recovered from fairly large samples [71], random fluctuations of uncertain significance were considered unimportant. The Begg and Mazmudar rank correlation test did not suggest across study bias/small study effects (Kendall’s score = 5; P > 0.05).

The number of studies on isolates (n > 10) recovered from each of animal and animal products by species, adoptees and of traveler origins were less than five; a disk (5μg) diffusion test does not reliably indicate Cip^ns^ [16], and higher breakpoint levels (> 0.06μg/mL) underestimate the proportion of Cip^ns^ strains. In addition, prior to 2011 a standardized International definition of MDR was not available [72], and MDR has not been uniformly defined in several studies other than those included in the meta-analyses. Accordingly, data from animals/animal products, adoptees, travelers and from studies with small sample sizes and/or methods that could likely introduce bias/heterogeneity [40–62, 64– 67], (S3 Table) were used to estimate relative and cumulative frequencies of mutations, PMQR genes, substituted amino acids, and mutant serotypes.

### Heterogeneity of phenotypic proportions

**Fig 3:**
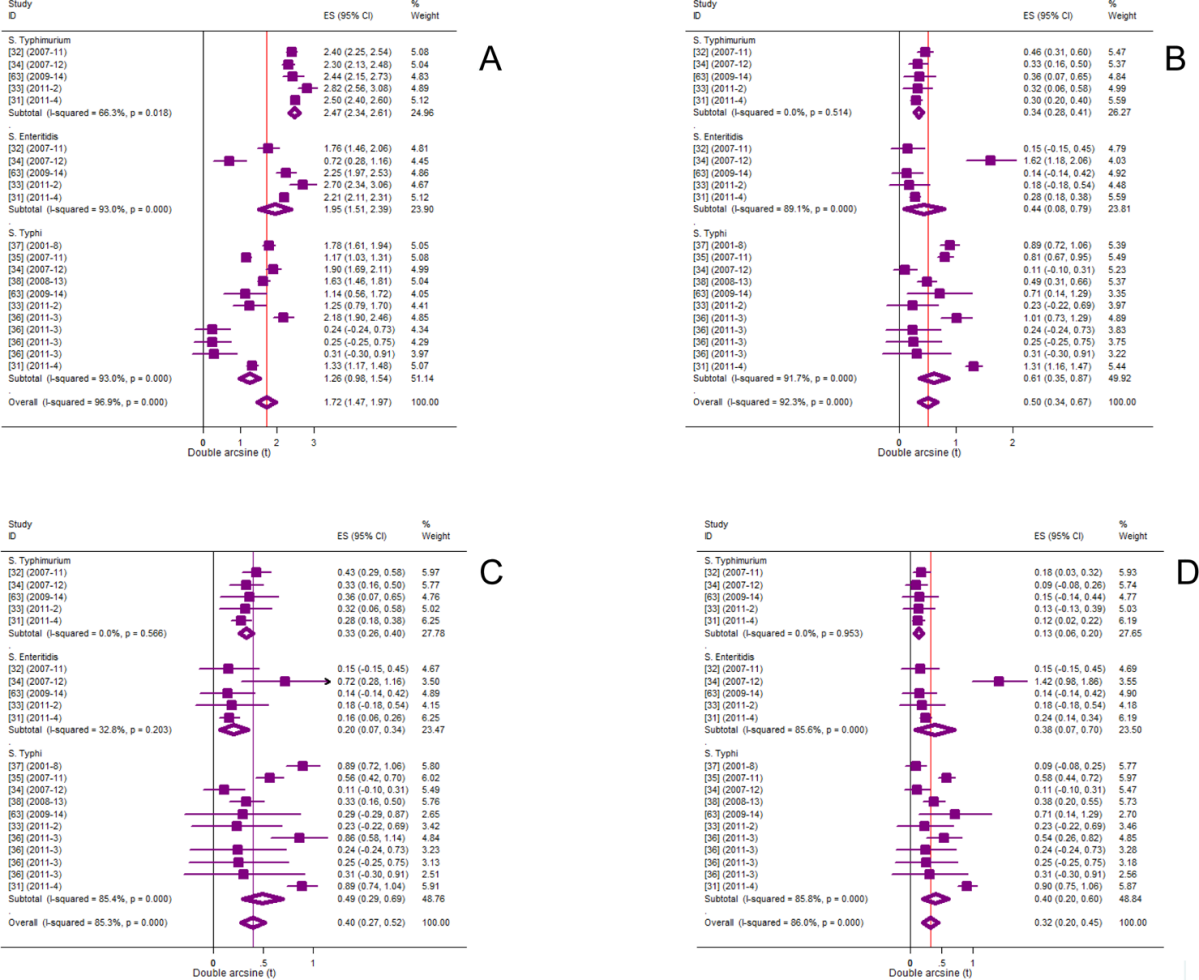
Forest plots of phenotypic proportions. MDR (A), Cip^ns^ (B), MDR-Cip^ns^ (C), nMDR-Cip^ns^ (D).

Fig 3 presents forest plots of proportions of MDR, and ciprofloxacin non-susceptible strains. The residuals of the heterogeneity estimates for each of MDR (P = 8E-94) and Cip^ns^ (P = 1E-22) invasive strains (S. Typhi, *S*. Typhimurium and *S*. Enteritidis) were highly significant and imply across-group differences. T^2^ estimates for MDR and Cip^ns^ strains, respectively, were 0.3171 and 0.1234, and those of MDR-Cip^ns^, and nMDR-Cip^ns^ were 0.0595 and 0.0628, respectively (Table 1).

**Table 1:** Heterogeneity of phenotypic proportions.

						I^2^ (95%CI)		
Phenotype	Serotype	N	K	Q	T^2^	I^2^	L	U
MDR	*S*. Typhimurium	800	5	12	0.0144	66	12	87
	*S*. Enteritidis	530	5	57	0.2249	93	87	96
	*S*. Typhi	836	11	143	0.1882	93	89	95
	Overall	2166	21	640	0.3171	97	96	98
Cip^ns^	*S*. Typhimurium	800	5	3	0.0000	0	0	75
	*S*. Enteritidis	530	5	37	0.1393	89	77	95
	*S*. Typhi	836	11	120	0.1558	92	87	95
	Overall	2166	21	261	0.1234	92	90	94
MDR-Cip^ns^	*S*. Typhimurium	800	5	3	0	0	0	72
	*S*. Enteritidis	530	5	6	0.0083	33	0	75
	*S*. Typhi	836	11	68	0.0828	85	76	91
	Overall	2166	21	136	0.0595	85	79	90
nMDR-Cip^ns^	*S*. Typhimurium	800	5	1	0	0	0	0
	*S*. Enteritidis	530	5	28	0.1011	86	68	94
	*S*. Typhi	836	11	70	0.0855	86	76	92
	Overall	2166	21	143	0.0628	86	80	90
Inv-salm	iNTS	47337	11	1196	0.031	99	99	99
	Typhoidal	47337	11	308	0.0078	97	96	98
	Overall	47337	11	1548	0.0178	99	98	99

Cip^ns^, ciprofloxacin non-susceptible; Inv-salm, invasive salmonellosis; K, number of studies; L, lower limit; MDR, multi-drug resistant; MDR-Cip^ns^, multi-drug resistant + ciprofloxacin non-susceptible; N, number of isolates; nMDR-Cip^ns^, non-multidrug resistant + ciprofloxacin non-susceptible; Q, Cochran’s-test; T^2^, tau squared; U, upper limit.

### Heterogeneity of genotypic proportions

**Fig 4:**
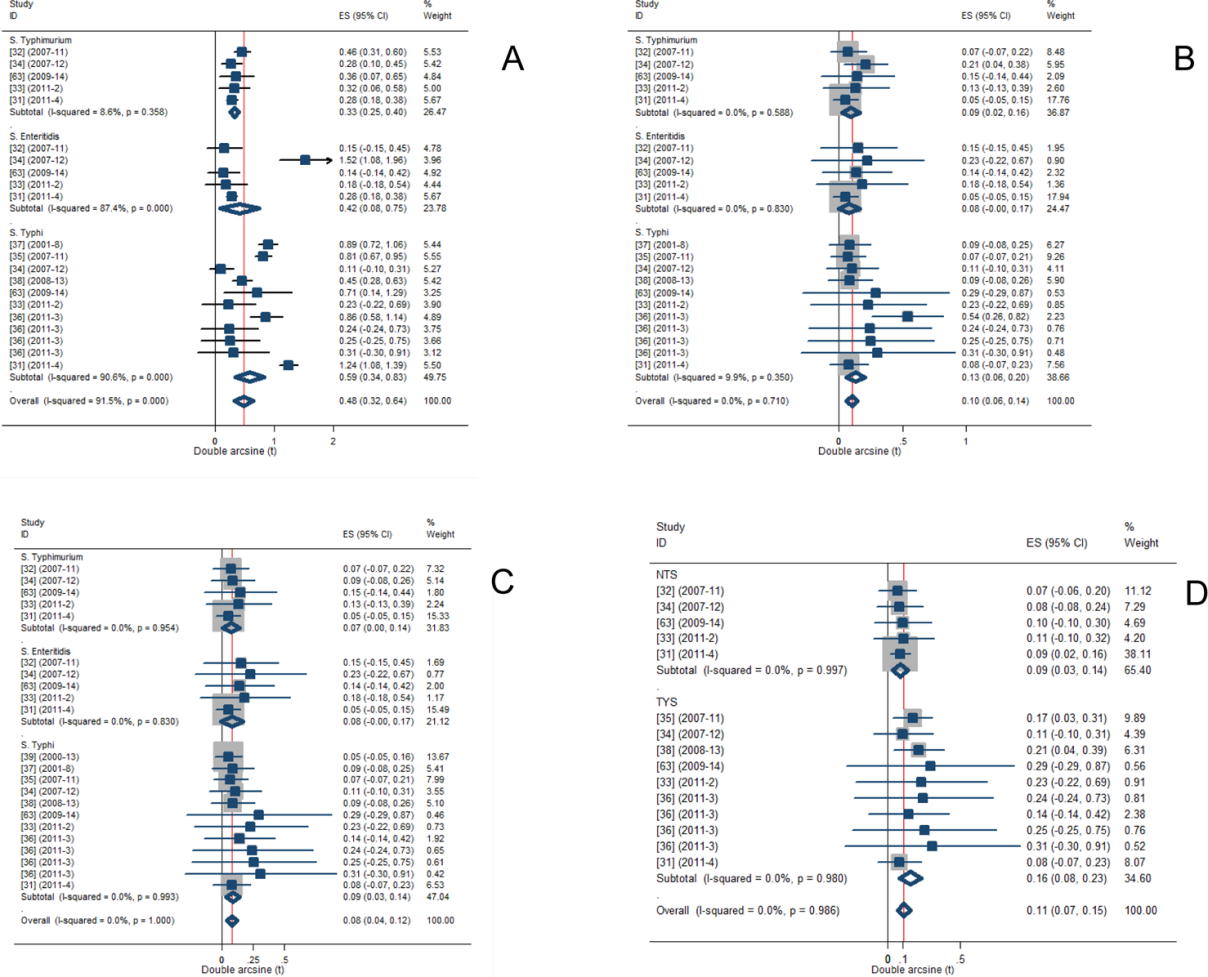
Forest plots of genotypic proportions. *gyrA* (A), *gyrB* (B), *parC* (C), *qnr* (*A*,*B*,*S*) (D).

Fig 4 presents forest plots of proportions of mutants, and strains with PMQR genes. The residual of the heterogeneity estimates of *gyrA* mutant typhoidal and non-typhi *Salmonella* was significant (P = 5E-21). The estimate was higher in *S*. Typhi (T^2^ = 0.137) than in NTS (T^2^ = 0.0259), and in *S*. Enteritidis than in *S*. Typhimurium (Table 2). The heterogeneity estimates for each of *gyrB* and *parC* mutants, and strains with PMQR genes (*qnrA*, *qnrB*, and *qnrC*) did not differ between typhoidal and NTS subgroups (P > 0.05).

**Table 2:** Heterogeneity of genotypic proportions.

						I^2^ (95%CI)		
Genotype	Serotype	N	K	Q	T^2^	I^2^	L	U
*gyrA*	*S*. Typhimurium	800	5	4	0.0007	9	0	81
	*S*. Enteritidis	830	5	35	0.1182	87	73	94
	*S*. Typhi	836	11	107	0.137	91	85	94
	Overall	2166	21	242	0.1105	92	88	94
*gyrB*	*S*. Typhimurium	800	5	3	0	0	0	71
	*S*. Enteritidis	530	5	1	0	0	0	44
	*S*. Typhi	836	11	11	0.0016	10	0	50
	Overall	2166	21	16	0	0	0	34
*parC*	*S*. Typhimurium	800	5	1	0	0	0	0
	*S*. Enteritidis	530	5	1	0	0	0	44
	*S*. Typhi	1180	12	3	0	0	0	0
	Overall	2510	22	5	0	0	0	0
*qnr(A*,*B*,*S)*	NTS	1330	5	0	0	0	0	0
	TYS	700	10	3	0	0	0	0
	Overall	2030	15	5	0	0	0	0

K, number of studies; L, lower limit; N, number of isolates; Q, Cochran’s-test

T^2^, tau squared; U, upper limit.

### Pooled phenotypic proportions

Pooled proportions of MDR and Cip^ns^ strains are given in Table 3. For each analysis, the single study omitted estimates including the 95%CIs lie within or closer to the 95% confidence bounds of the respective pooled estimate. The proportion of MDR *S*. Typhimurium was higher than the proportion of MDR *S*. Typhi (OR = 15.6, 95%CI = 11.8, 20.5). Conversely, the proportion of Cip^ns^ *S*. Typhi was higher than the proportion of Cip^ns^ *S*. Typhimurium (OR = 3.3, 95%CI = 2, 5.5). The proportions of MDR-Cip^ns^ and nMDR-Cip^ns^ isolates, respectively, were estimated at 3.8% (95%CI = 1.8, 6.5) and 2.7% (95%CI = 0.9, 5.3) (Table 3). Of 785 isolates tested with both ciprofloxacin and nalidixic acid [32, 33, 35, 37,63–65], 70(8.9%) were non-susceptible to both drugs.

**Table 3:** Pooled phenotypic proportions.

				P (95%CI)			
Phenotype	Serotype	N	K	P	L	U	OR (95%CI)
MDR	*S*. Typhimurium	800	5	89.3	84.8	93.1	Tp vs.Ty; 15.6 (11.8,20.5)
	*S*. Enteritidis	530	5	68.5	47.1	86.6	
	*S*. Typhi	836	11	34.8	22.3	48.5	
	Overall	2166	21	57.4	44.9	69.5	
Cip^ns^	*S*. Typhimurium	800	5	2.9	1.8	4.1	Ty vs. Tp; 3.3 (2,5.5)
	*S*. Enteritidis	530	5	4.6	0.1	14.8	
	*S*. Typhi	836	11	8.9	3	17.6	
	Overall	2166	21	6.2	2.8	10.7	
MDR-Cip^ns^	*S*. Typhimurium	800	5	2.6	1.6	3.9	
	*S*. Enteritidis	530	5	1	0	2.8	
	*S*. Typhi	836	11	5.9	2.1	11.4	
	Overall	2166	21	3.8	1.8	6.5	
nMDR-Cip^ns^	*S*. Typhimurium	800	5	0.4	0	1	
	*S*. Enteritidis	530	5	5.2	0	17.9	
	*S*. Typhi	836	11	3.5	0.7	8.4	
	Overall	2166	21	2.7	0.9	5.3	
Inv-salm	iNTS	47337	11	1.9	0.7	3.6	NTS vs. TyS; 1.1(0.98,1.2)
	Typhoidal	47337	11	1.7	1.1	2.5	
	Overall	47337	11	1.8	1.1	2.6	

Cip^ns^; ciprofloxacin non-susceptible; Inv-salm, invasive salmonellosis; K, number of studies; L, lower limit; MDR, multi-drug resistant; MDR-Cip^ns^; multi-drug resistant + ciprofloxacin non-susceptible; N, number of isolates; nMDR-Cip^ns^; non-multidrug resistant + ciprofloxacin non-susceptible; NTS, non-typhoidal *Salmonella*; OR, odds ratio; P, pooled proportion; Q, Cochran’s-test; T^2^, tau squared; Tp, *S*. Typhimurium; Ty, *S*. Typhi; TyS, typhoidal *Salmonella*; U, upper limit.

### Pooled genotypic proportions

The pooled proportions of mutants and strains with PMQR genes are given in Table 4. All single study omitted estimates including the 95%CIs lie within or closer to the 95% confidence bounds of the respective means. Mutations at *gyrA*, *gyrB and parC* but *parE* have been identified (Table 4). The overall pooled proportion of *gyrA* mutants was estimated at 5.7% (95%CI = 2.6, 9.8). The proportion of *gyrA* mutants was higher in typhoidal than in non-typhi *Salmonella* (OR = 2.9, 95%CI = 1.9, 4.4), and in *S*. Typhi than in *S*. Typhimurium (OR = 3.3, 95%CI = 2, 5.7). The proportions of *gyrB* and *parC* mutants, and strains with PMQR genes (*qnrA*, *qnrB* and *qnrS*), respectively, were estimated at 0.2% (95%CI = 0.1, 05), 0.1% (95%CI = 0, 3), and 0.3% (95%CI = 0.1, 0.6).

**Table 4:** Pooled genotypic proportions.

				P (95%CI)			
Genotype	Serotype	N	K	P	L	U	OR (95%CI)
*gyrA*	*S* Typhimurium	800	5	2.6	1.6	4	
	*S*. Enteritidis	530	5	4.2	0.1	13.2	
	*S*. Typhi	836	11	8.3	2.8	16.2	Ty vs. Tp; 3.3 (2,5.7)
	Overall	2166	21	5.7	2.6	9.8	
*gyrB*	*S*. Typhimurium	800	5	0.2	0	0.6	
	*S*. Enteritidis	530	5	0.1	0	0.6	
	*S*. Typhi	836	11	0.4	0	1	
	Overall	2166	21	0.2	0.1	0.5	
*parC*	*S* Typhimurium	800	5	0.1	0	0.5	
	*S*. Enteritidis	530	5	0.1	0	0.6	
	*S*. Typhi	1180	21	0.2	0	0.5	
	Overall	2510	21	0.1	0	0.3	
*qnr(A*,*B*,*S)*	iNTS	1330	5	0.1	0	0.4	
	*S*. Typhi	700	10	0.5	0.1	1.2	
	Overall	2030	15	0.3	0.1	0.6	

Cip^ns^, ciprofloxacin non-susceptible; K, number of studies; L, lower; N, number of isolates; OR, odds ratio; P, pooled proportion; Tp, *S*. Typhimurium; Ty, *S*. Typhi; U, upper limit.

### Pooled proportion of *gyrA* mutant invasive infections

**Fig 5:**
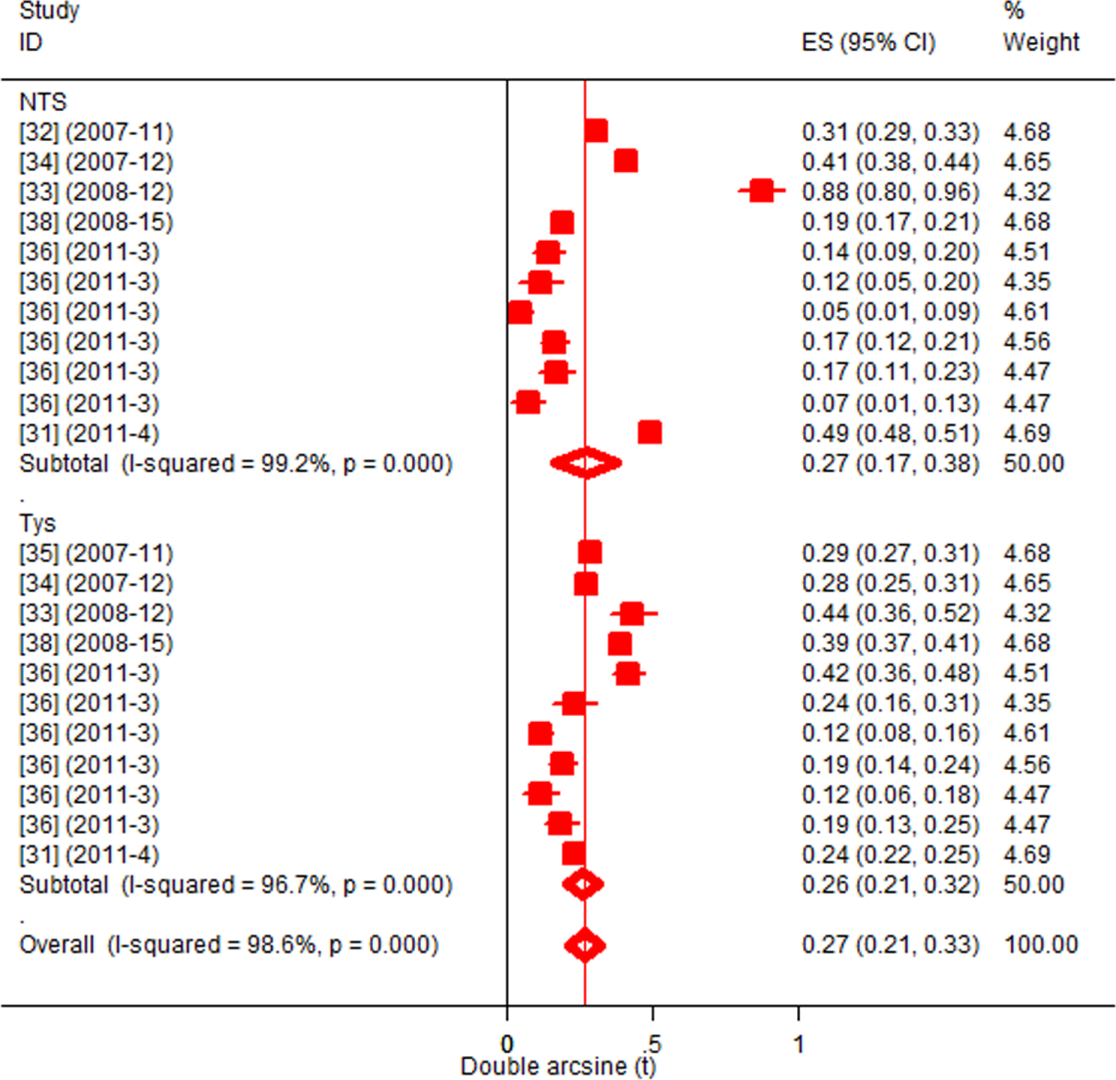
Forest plot of proportions of invasive *Salmonellosis*.

Fig 5 presents the occurrence of invasive *Salmonella* (typhoidal and non-typhi) disease in SSA. The estimates were based on data from nine countries [31–36, 38]. The overall pooled proportion was estimated at 1.8% (95%CI = 1.1, 2.6). The proportions of iNTS disease (1.9%, 95%CI = 0.7, 3.6) and typhoidal infections (1.7%, 95%CI = 1.1, 2.5) did not differ (P > 0.05). The pooled proportion of infections with *gyrA* mutants was estimated at 0.1% (95%CI = 0.03, 0.26).

### Preponderance of gene mutations

Relative and cumulative frequencies of gene mutations in quinolone resistant Salmonellae are given in Table 5. Mutations at *gyrA* were the most frequent (82%, 95%CI = 78, 86). Of these, 92.8% (256) were recovered from human patients who sought medical attention and three asymptomatic adopted children. Mutations at *gyrB* and *parC*, respectively, were identified in 2.9% (95%CI = 1.5, 5.6) and 26.6% (95%CI = 21.9, 31.8) of the isolates tested for each genotype. No *parE* mutation has been reported.

**Table 5:** Relative and cumulative frequencies of gene mutations.

					P (95%CI)^‡^			
Gene	Host	Group	Setting	N	P	L	U	References
*gyrA*	Human	NTS	Cm, Ad	94	77.7	68.2	84.9	[31–34,45–49,51–54,63]
	Human	TyS	Cm	162	84	77.5	88.8	[31,35–38,41–43,63,64]
	Human	NTS	Tr	47	100	92.4	100	[54–56]
	Animal	NTS	A/P	33	60.6	43.7	75.3	[52, 55,56,60]
			Overall	336	82.1	77.7	85.9	[31–38, 41–43, 45–49, 51–56,63,64]
*gyrB*	Human	NTS	Cm, Ad	62	4.8	1.7	13.3	[31–34,46,48,49,51,52,54,63]
	Human	TyS	Cm	137	2.2	0.7	6.2	[31,35,36,38,41–43,63,64]
	Human	NTS	Tr	47	0	0	7.6	[54–56]
	Animal	NTS	A/P	32	6.3	1.7	20.1	[52,55,56]
			Overall	278	2.9	1.5	5.6	[31–36,38, 41–43, 46,48,49, 51–56,63,64]
*parC*	Human	NTS	Cm, Ad	63	22.2	13.7	33.9	[31–34,46,48,49,51–54,63]
	Human	TyS	Cm,	162	4.9	2.5	9.4	[31,35–38,41–43,63,64]
	Human	NTS	Tr	47	87.2	74.8	94	[54–56]
	Animal	NTS	A/P	33	54.5	38	70.2	[52,55,56,60]
			Overall	305	26.6	21.9	31.8	[31–36,38,41–43,46,48,49,51–56,60,63,64]
*parE*	Human	NTS	Cm, Ad	30	0	0	11.4	[34,48,49,51,52,54]
	Human	TyS	Cm	68	0	0	5.3	[36,37,38,41–43]
	Human	NTS	Tr	47	0	0	7.6	[54–56]
	Animal	NTS	A/P	32	0	0	10.7	[52,55,56]
			Overall	177	0	0	2.1	[34,36–38,41–43,48,49,51,52,54–56]

Ad, adoptees; A/P, animals/animal products; Cm, community; P, proportion; L, lower limit; N, number; NTS, non-typhoidal *Salmonella*; OR, odds ratio; Tr, travel-associated; TyS, typhoidal *Salmonella*; U, upper limit.

^‡^Score confidence interval.

### Preponderance of mutated codons

**Fig 6:**
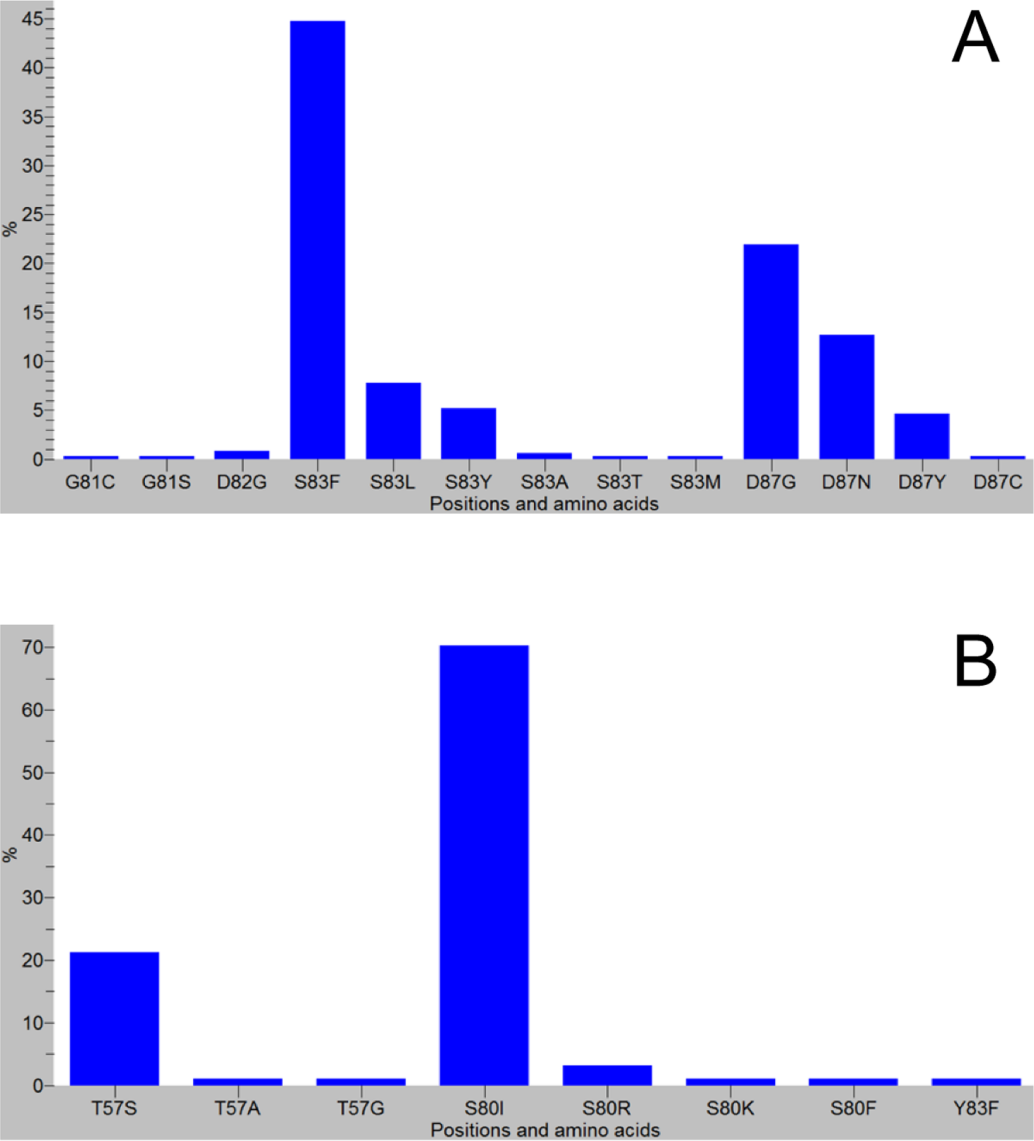
Mutated codons and substituted amino acids. *gyrA*, (A); *parC*, (B).

The most frequently mutated codons in the QRDR of *gyrA* were codons for Ser83 (60%, 204/346) and Asp87 (39.6%, 137/346) (Fig 6). Other *gyrA* codons included codons encoding Gly81, Asp82, Ala119 [42] and Glu133 [35, 36, 63]. At *parC*, mutations most frequently occurred at codons encoding Ser80 followed by Thr57, and most have been identified in association with *gyrA* mutations (Ser83 or Asp87).

### Preponderance of substituted amino acids

The amino acids the most frequently substituted at GyrA were phenylalanine (155/204) and leucine (27/204) at Ser83, and glycine (76/137) and asparagine (44/137) at Asp87 (Table 6). The amino acids the most frequently substituted at positions Ser80 and Thr57 of ParC, respectively, were isoleucine (66/71) and serine (20/22).

**Table 6:** Frequencies of substituted amino acids.

			Substituted amino acids															
Enzyme	Position	n_p_	Phe	Leu	Tyr	Thr	Gly	Asn	Cys	Asp	Ile	Ser	Arg	Lys	Ala	Met	His	References
GyrA	Ser83	204	155	27	18	1	-	-	-	-	-	-	-	-	2	1	-	^‡^R
(n_s_ = 276)	Asp87	137	-	-	16	-	76	44	1	-	-	-	-	-	-	-	-	^†^R
	Asp82	3	-	-	-	-	3	-	-	-	-	-	-	-	-	-	-	[42]
	Gly81	2	-	-	-	-	-	-	1	-	-	1	-	-	-	-	-	[33, 42]
GyrB	Ser464	3	3	-	-	-	-	-	-	-	-	-	-	-	-	-	-	[36]
(n_s_ = 8)	Ser463	1	-	-	-	-	-	-	-	-	-	-	-	-	1	-	-	[52]
	Glu466	2	-	-	-	-	-	-	-	2	-	-	-	-	-	-	-	[34,49]
	Val423	2	-	-	-	-	2	-	-	-	-	-	-	-	-	-	-	[52]
	Asp459	2	-	-	-	-	-	-	-	-	-	-	-	-	-	-	2	[52]
ParC	Ser80	71	1	-	-	-	-	-	-	-	66	-	3	1	-	-	-	[42,49,52–56]
(n_s_ = 81)	Thr57	22	-	-	-	-	1	-	-	-	-	20	-	-	1	-	-	[36,42,46,52,60]
	Tyr83	1	1	-	-	-	-	-	-	-	-	-	-	-	-	-	-	[52]

Ala, alanine; Arg, arginine; Asn, asparagine; Asp, aspartic acid; Cys, cystine; Gly, glycine; His, histidine; Ile, isoleucine; Leu, leucine; Lys, lysine; Met, methionine; n_p_, number of mutations by position; n_s_, number of strains; Phe, phenylalanine; R, references; Ser, serine; Thr, threonine; Tyr, tyrosine.

^‡^R, [31,33–38,42,46–48,52–56,64]

^†^R, [31,32,34,35,42,43,47,48,52–56,60,63]

### Preponderance of PMQR genes

Of the PMQR genes, *qnr*A, *qnr*B, *qnr*S, *aac(6′)-Ib-cr* and *qep*A but *qnr*C (0/9 4) [31, 40, 62, 65], *qnrD* (0/203) [31, 38, 40, 43, 48, 49, 51, 52, 54–56, 62, 65] and *oqxA/B* (0/85) [31,55] have been identified (Table 7). The proportions of strains with *qnr*A, *qnr*B, *qnr*S, and *aac(6′)-Ib-cr* genes, respectively, were 1.5% (95%CI = 0.8, 2.9), 5.9% (95%CI = 4.2, 8.3), 3.1% (95%CI = 1.9, 5.1), and 4.2% (95%CI = 2.6, 6.6). Most isolates with PMQR genes were NTS.

**Table 7:** Frequencies of strains with PMQR genes.

					P (95%CI)^‡^			
Genotype	Host	Group	Setting	N	P	L	U	References
*qnrA*	Human	NTS	Cm, Ad	105	5.7	2.6	11.9	[31–34,44–52,54,63,65,66]
	Human	TyS	Cm	159	0	0	2.4	[31,35–38,42,43,63,64]
	Human	NTS	Tr	48	0	0	7.4	[54–56,65]
	Animal	NTS	A/P	223	0.9	0.2	3.2	[40,52,55–62]
			Overall	535	1.5	0.8	2.9	[31–38,40,42–52,54–56,63–66]
*qnrB*	Human	NTS	Cm, Ad	76	19.7	12.3	30	[31–34,45,46,48–52,54,63,65,66]
	Human	TyS	Cm	159	0	0	2.4	[31,35–38,42,43,63,64]
	Human	NTS	Tr	48	2.1	0.4	10.9	[54–56,65]
	Animal	NTS	A/P	223	6.3	3.8	10.3	[40,52,55–62]
			Overall	506	5.9	4.2	8.3	[31–38,40,42–52,54–56,63–66]
*qnrS*	Human	NTS	Cm, Ad	76	0	0	4.8	[31–34,45,46,48–52,54,63,65,66]
	Human	TyS	Cm	134	1.5	0.4	5.3	[31,35,36,38,42,43,63,64]
	Human	NTS	Tr	48	0	0	7.4	[54–56,65]
	Animal	NTS	A/P	223	5.8	3.4	9.7	[40,52,55–62]
			Overall	481	3.1	1.9	5.1	[31–38,40,42–52,54–56,63–66]
*aac(6′)-Ib-cr*	Human	NTS	Cm, Ad	45	17.8	9.3	31.3	[31,45,48–52,54,65]
	Human	TyS	Cm	95	0	0	3.9	[31,37,38,43]
	Human	NTS	Tr	48	0	0	7.4	[54–56,65]
	Animal	NTS	A/P	219	4.1	2.2	7.6	[52,55–59,61,62]
			Overall	407	4.2	2.6	6.6	[31,37,38,43,45,48–52,54–59,61,62,65]
*qepA*	Human	NTS	Cm, Ad	49	0	0	7.3	[31,34,48,49,51,52,54,65]
	Human	TyS	Cm	82	0	0	4.5	[31,36,38,43]
	Human	NTS	Tr	48	0	0	7.4	[54–56,65]
	Animal	NTS	A/P	168	0.6	0.1	3.3	[52,55,56,59,61]
			Overall	347	0.3	0.1	1.6	[31,34,36,38,43,48,49,51,52,54–56,61,65]

Ad, adoptees; A/P, animals/animal products; Cm, community setting; L, lower limit; N, number; P, proportion; Tr, travel associated; U, upper limit.

^‡^Score confidence interval.

### Mutant serotypes

**Fig 7:**
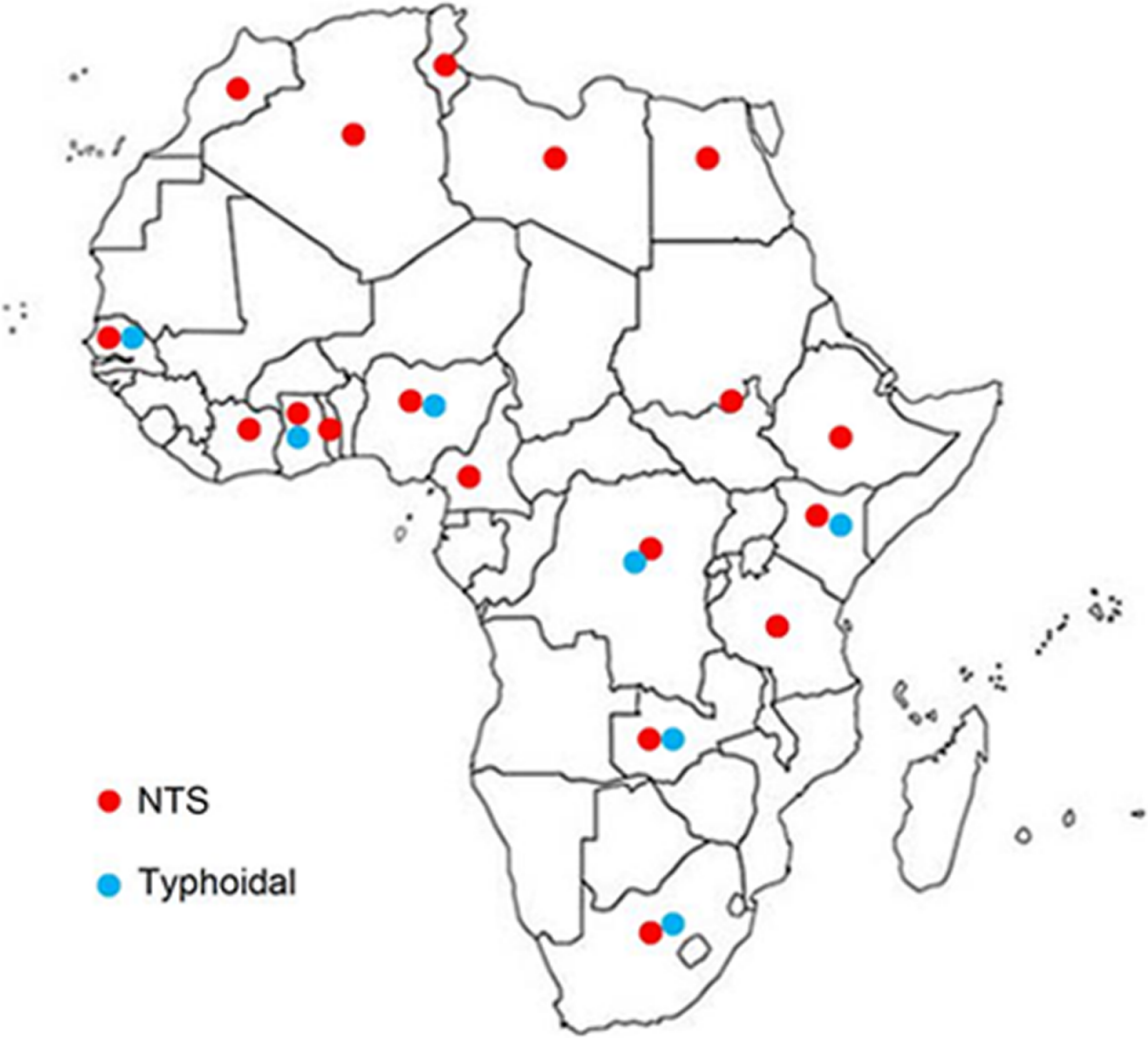
Distribution of reported *gyrA* mutants. [http://www.worldatlas.com/webimage/countrys/af.htm].

A distribution map of reported *gyr*A mutant typhoidal and non-typhoidal *Salmonella* is given in Fig 7. Twenty-three mutant serotypes have been identified (Table 8). *S*. Typhi was the most frequently reported (139/287) followed by *S*. Kentucky (69/287), *S*. Typhimurium (34/287) and *S*. Enteritidis (16/287). The numbers of mutations differed between typhoidal and NTS subgroups. Most double (9/10), triple (56/59) and quadruple (10/11) mutants were NTS. Most triple (47/63) and quadruple (10/63) mutants were *S*. Kentucky strains [52–56].

**Table 8:** Reported occurrence of mutant serotypes.

Regions	Source	Serotype	N	*gyrA*	*gyrB*	*parC*	References
ECWN	Human	*S*. Typhi	139	135	3	7	[31, 35–38,42,43,63,64]
EWN	H/A	*S*. Kentucky	69	69	-	63	[52–56]
CWS	Human	*S*. Typhimurium	34	32	2	-	[31–34,47,49,63]
CW	Human	*S*. Enteritidis	16	16	-	-	[31,34]
Southern	Human	*S*. Isangi	7	7	-	-	[47]
Eastern	Human	*S*. Concord	4	3	-	4	[46]
Southern	Human	*S*. Senftenberg	2	2	-	2	[48]
Eastern	Animal	*S*. Livingstone	2	2	-	-	[52]
Southern	Human	*S*. Kissi	1	1	-	-	[47]
Southern	Human	*S*. Kivu	1	1	-	-	[47]
Western	Human	*S*. Paratyphi A	1	1	-	1	[36]
Southern	Human	*S*. Reading	1	1	-	-	[47]
Central	Human	*Salmonella* 4,5	1	1	-	-	[32]
Southern	Human	Untypable	1	1	-	-	[47]
Eastern	Human	V:ROUGH-O;-:-	1	-	1	-	[52]
Eastern	Animal	I:6;7,14:-:I,w	1	1	-	-	[52]
Eastern	Animal	*S*. Agona	1	-	-	1	[52]
Eastern	Animal	*S*. Braenderup	1	-	1	1	[52]
Northern	Animal	*S*. Hadar	1	1	-	1	[60]
Eastern	Animal	*S*. Haifa	1	1	-	-	[52]
Eastern	Animal	*S*. Miami	1	-	-	1	[52]
Eastern	Animal	*S*. Mikawasima	1	-	1	-	[52]
Eastern	Animal	*S*. Virchow	1	1	-	-	[52]

CW, Central and Western; CWS, Central, Western and Southern; ECWN, Eastern, Central, Western and Northern; EWN, Eastern, Western and Northern.

### Ciprofloxacin MICs and genetic determinants

Two levels of resistance (≤ 1μg/mL and ≥ 4μg/mL) were identified (Table 9). The highest MICs (4μg/mL to > 32 μg/mL) have been recorded in triple mutant *S*. Senftenberg [48], and *S*. Kentucky [53–56]. In other serotypes but three NTS with PMQR genes but no mutations [51], the MICs were ≤ 1μg/mL. In quadruple mutant *S*. Kentucky (*gyrA*-Ser83Phe and Asp87Gly, and *parC-*Ser80Ile and Thr57Ser), zone diameter (ZD) decreases of two to four-fold (8-14mm) were recorded [52].

**Table 9:** Ciprofloxacin MICs and genetic determinants.

Serotype	N	*gyrA*	*gyrB*	*parC*	NM	PMQR	MIC (μg/mL)	References
*S*. Typhi	48	0	0	0	0	nd	0.25–0.38	[41]
NTS	6	0	0	0	0	+	0.08–2	[51]
*S*. Typhi	126	123	3	0	1	+/-	>0.06–1	[31,35–37,43,63,64]
*S*. Typhi	7	6	0	7	1–4	-	0.125–0.5	[42]
*S*. Enteritidis	16	16	0	0	1	+/-	>0.06–0.5	[31,34]
*S*. Kentucky	6	6	0	0	1	-	0.125–0.5	[56]
*S*. Kentucky	53	53	0	53	3	-	4- >32	[53–56]
*S*. Senftenberg	2	2	0	2	3	+	>4	[48]
*S*. Senftenberg	1	0	0	0	0	+	≥0.12	[65]
*S*. Typhimurium	21	20	1	0	1	+/-	>0.06–0.5	[31–34, 49,63]
*S*. Concord	4	3	0	4	1,2	+/-	0.5–1	[46]
*S*. Concord	3	0	0	0	0	+	0.12–0.5	[65]

## Discussion

Here, we describe the heterogeneity and preponderance of FQ-resistance genetic determinants in *Salmonella* isolates of Africa. Most invasive infections have been due to *S*. Typhimurium, *S*. Enteritidis and *S*. Typhi. This is consistent with reports of previous reviews/meta-analyses on the occurrence of salmonellosis in SSA [3, 4, 27]. The heterogeneity estimates demonstrate variability in the occurrence of MDR, CIp^ns^, and *gyrA* mutants, but parallel occurrence of each of *gyr*B and *par*C mutants, and strains with PMQR genes in both typhoidal and non-typhi *Salmonella*.

The substantial inconsistency estimates imply potential variations in the distributions of MDR and FQ-resistant invasive strains across locations. For instance, the *S*. Typhi H58 lineage is frequently associated with MDR and FQ-resistance and reportedly predominate in Eastern and Southern Africa, but is rare in other regions [39]. By the same token, despite MDR non-typhi *Salmonella* occurs extensively [73], most iNTS diseases have been associated with two *S*. Typhimurium ST313 lineages–I and II [74]. These lineages emerged with MDR acquisition, and a chloramphenicol driven clonal replacement of lineage I by lineage II, and dispersal of the latter from the Central to other regions have been postulated [75,76]. Similarly, two *S*. Enteritidis clades (West African and Central/East African) resistant to one or more antimicrobial classes (1–4) have been implicated in iNTS disease [77]. Nevertheless, the considerable differences in the occurrence of iNTS disease (e.g. Central –1186/24370, Eastern- 9/4407), (Fig 4) suggest variability in the distribution of these lineages (*S*. Typhimurium and *S*. Enteritidis) across regions and countries, and locations within a country. Similarly, a systematic review [4], and a recent multi-country based study [78] have reported variability in the occurrence of iNTS disease and/or typhoidal infection in SSA.

The variability in drug resistance might essentially have been due to differences in the pattern and frequency of use of antimicrobials across locations and time. In resource-poor settings, antimicrobial self-medication is widespread and has been linked with inappropriate use [79]. Elsewhere in Asia, the convergence of poor public health system, non-prescription antimicrobial use, and rising income has been implicated as a factor that created favorable conditions for the selection and spread of drug resistant pathogens [80]. Antimicrobial use has also been associated with persisting AMR (up to a year) at the individual level [81], and resistant bacteria are common in communities with frequent non-prescription use [82]. In areas where typhoid fever is endemic, most patients self-treat [10], and non-prescription antimicrobial use as high as 100% has been documented in Eastern and Western Africa [82]. The extent of the problem could, however, differ across countries and within a country (urban/rural) depending on drug availability. For instance, in Kenya the FQs have been used since the last quarter of the 1990s, and of the *S*. Typhi isolated in 2000–2, 47% (48/102) were ciprofloxacin non-susceptible [41]. Likewise, in Nigeria the use of FQs was associated with an increase in the occurrence of FQ-resistant *Escherichia col*i between 2005 and 2009 [83]. By contrast, among NTS recovered from human samples in 2013/4 in Ethiopia, the occurrence of ciprofloxacin non-susceptible strains but two *S*. Kentucky and one V:ROUGH-O;-:- was low [52]. In general, the *gyrA* region is under a strong positive selection pressure, and mutations have reportedly occurred independently on multiple occasions [39]. Although FQ-resistant *S*. Typhi H58 emerged in Asia and disseminated globally–including Eastern and Southern Africa [39]–invasive *S*. Typhimurium [75, 76] and *S*. Enteritidis [77] originated in Africa, and are common causes of disease in Africa. Taken together, the AMR profiles (MDR-Cip^ns^ and nMDR-Cip^ns^) of both typhoidal and iNTS isolates point towards a more than likely locally induced non-quinolone and quinolone based selection of FQ-resistant strains.

Most ciprofloxacin non-susceptible (Cip^ns^) strains were nalidixic acid non-susceptible (Nal^ns^). However, there have been reports of strains with the Nal^s^Cip^ns^ phenotype [36, 41, 43, 47, 51] with most being from Kenya [41]. This atypical phenotype has also been identified in *S*. Typhi [84, 85] and NTS [14] elsewhere. In India, Nal^s^ and Nal^ns^ phenotypes, respectively, were reported to have predictive values of 95% for Cip^s^, and 36% for Cip^ns^ in S. Typhi [86]. Although reports on the distribution of strains with the Nal^s^Cip^ns^ phenotype are scarce, such strains could occur in several countries in Africa.

Mutations at Ser83 and Asp87 of *gyrA* were the most frequently identified, and appear to account for FQ-non-susceptibility in most strains. The occurrence of Glu133Gly mutation concomitantly with *gyrA* (Ser83 and Asp87) [35, 36, 63] and *gyrB* mutations [36], and in Nal^s^ and/or Cip^s^ [34, 35] strains implies a no association with FQ-resistance. In resistant isolates, mutations frequently occur between positions 67 and 106 [87], and in a sequence analysis of GenBank data, while 99.1% (212/214) of the NTS sequences carried the Glu133 residue, 96% (97/101) of the *S*. Typhi did not have it [88]. Furthermore, compared to *gyrA* mutations the occurrence of *gyrB* and *parC* mutations were low, and the relative contributions of these mutations to FQ-non-susceptibility in strains commonly implicated in invasive salmonellosis may be low. Among a global collection of *S*. Typhi, the *gyrB* mutant proportion was 2.2% (42/1832), and *parC* coding changes were detected in 1.6% (29/1832) with almost all (28/29) belonging to the H58 lineage [39]. The overall data demonstrate *gyrA* mutation as the foremost determinant of FQ-resistance, and Ser83 and Asp87 as the major mutation ‘hot spots’.

The estimates of the occurrence of salmonellosis in SSA [3, 4, 89, 90] differ, and underestimation of the incidence of iNTS disease but overestimation of that of typhoid fever was proposed [91]. Accordingly, to put the proportion of *gyrA* mutant infections in perspective, we estimated the proportion of invasive salmonellosis. As a result, the proportions of patients infected with iNTS and typhoidal strains were comparable, and the prevalence of infection with *gyrA* mutants was 0.1% (Table 3). The proportion of iNTS disease in SSA (1.9%) was in accord with the estimate of Reddy et al. [27] (~2%, 960/(58296–10230))–authors’ calculations after excluding data from North Africa where 99% of the isolates were *S*. Typhi). Nevertheless, as these estimates were not derived from population-based data, the true prevalence and incidence of invasive salmonellosis and thus infections with *gyrA* mutants could be higher. Furthermore, the estimates denote likely higher incidence rates than were estimated for iNTS disease ((227(152–341)/100000) [3], 0 to 237 (178–316) per 100000 person years [78]) and typhoidal infections ((10-100/100000) [89], (0 to 383 (274–535) per 100000 person years [78]). The differences in the estimated occurrence of the disease (prevalence/incidence) might have originated from differences in study periods, countries, locations, and numbers of study participants.

Ciprofloxacin was the test FQ, and data on other FQs is not available. However, the varied amino-acid substitutions at mutation positions suggest potential cross-resistance to other FQs. Lower binding of Ser83→Phe/Tyr to nalidixic acid, and Asp87→Gly/Tyr to ciprofloxacin [92], Ser83→Phe/Leu substitution to ciprofloxacin resistance but not to levofloxacin [93], Ser83→Tyr/Ile substitution with resistance to both ciprofloxacin and levofloxacin [93], and Ser83Phe + Asp87Val of *gyrA* + *parC* mutations with resistance to ciprofloxacin, ofloxacin, levofloxacin and gatifloxacin [94] have been reported. In *Escherichia coli*, similarities in drug structures and genotypes were proposed as factors accounting for parallel ciprofloxacin and norfloxacin MICs, and levofloxacin and gatifloxacin MICs [95].

The occurrence of PMQR genes was generally low, and comparable between isolates of human and animal origins. Reports show regional differences in the occurrence of PMQR genes. For instance, in the USA PMQR genes were reported to have been rare in *Salmonella* [96]. In contrast, among *Salmonella* collected from 13 countries in Europe, PMQR genes were detected in 59% (288/485) with *qnrB* being predominant (28.5%, 138/485) followed by *qnrS* (26%, 125/485) and *qnrD* (4.5%, 22/485) [97]. In a global analysis of reports on 20,960 enteric bacterial isolates, the prevalence of *qnrA*, *qnrB*, *qnrS*, and *aac (6’)-Ib-cr* was estimated at 1.5%, 4.6%, 2.4%, and 10.8%, respectively [68]. In Asia, higher occurrence of PMQR genes in enteric bacteria of human (65%, 414/642) [98] and animal origins (57%, 282/495) [99] have been reported. In general, PMQR genes reportedly provide low-level resistance that does not exceed the clinical breakpoint level [100], and the wide MIC ranges have been attributed to differences in plasmid copy number and gene expression [101].

Of the 23 mutant serotypes, triple and quadruple mutations have commonly been detected in *S*. Kentucky. This serotype has been identified from various samples collected from several countries in Africa and elsewhere [52–56, 102]. Whilst most *S*. Kentucky isolated in the earlier years were triple mutants [54–56], strains isolated from humans and domestic animals in 2013/4 in Ethiopia (n = 10) [52] were quadruple mutants. The data from Ethiopia is suggestive of the proclivity of *S*. Kentucky strains to mutate, and the distribution of the serotype across a range of hosts, countries and regions [52–56] implies its evolutionary success similar to *S*. Typhi H58 that reportedly has disseminated globally [39]. Furthermore, there have been reports of strains (ST198-X1) with genes that confer resistance to the cephalosporins (CMY-2, CTX-M-15 and VEB), carbapenemes (OXA-48 and VIM-2) and azithromycin (*mphA*) [53, 54]. Accordingly, the mutability, occurrence in various animal hosts and resistance to antimicrobials critically important for human health are reminiscent of the danger *S*. Kentucky strains might pose in the near future. A recent risk assessment study on Antibiotic Pan Drug Resistance (PDR) in the UK has estimated 284,000 cases of PDR Gram-negative bacteraemia leading to 79,000 deaths in a span of 20 years [103].

The ciprofloxacin MICs and associated genetic determinants depict a complex association between FQ-resistance phenotype and genotype, and what each determinant accounts for is indistinct (Table 9). This complexity might be due to effect modifying or confounding factors and lurking variables. In all reports included in this study, efflux and influx associated genetic mechanisms have not been addressed. A regression analysis could not be performed because of the limited number of studies and co-variable differences among studies. However, the frequent co-occurrence of target alterations and efflux activation [104], and the crucial role of the efflux system for the development of high-level resistance to FQs [101] have been reported. In *Salmonella*, AcrAB is the major multidrug efflux pump and RamA is a positive regulator of the *acrAB* transcription [105]. Nevertheless, the role of the efflux system in high-level quinolone resistant serotypes/strains has not been consistently described. For instance, a South African study on *S*. Typhi mutants has reported a 16- to 32- fold decrease in nalidixic acid MIC, and a 2 to 8- fold decrease in ciprofloxacin MIC in the presence of an efflux pump inhibitor [42]. By contrast, a study on *S*. Typhi and *S*. Paratyphi A strains of diverse origins, and with varying levels of resistance to nalidixic acid (16–1024 μg/mL) and ciprofloxacin (0.125–8 μg/mL) has reported the absence of mutations associated with the AcrAB efflux system genes [106]. Furthermore, although *ramR* mutations were associated with ciprofloxacin resistance in *S*. Typhimurium [107,108], in *S*. Kentucky, these mutations were recorded in only three of the 27 strains examined [109]. Accordingly, the AcrAB efflux system associated gene mutations do not appear to provide a general explanation for the wide MIC range recorded in *S*. Kentucky strains (4 to >32μg/mL) [53–56]. Overall, the data suggest potential interactions of the determinants of resistance (antagonistic, additive or synergistic) or mechanisms that have not yet been described.

### Limitations, bias and strength of evidence

We have adhered to the PRISMA statement for reporting systematic reviews and meta-analyses [21]. However, as is the case in systematic reviews/meta-analyses, this study has limitations. First, the number of studies was limited; data from North Africa was scarce, and a region-based analysis was not done. Second, although we retrieved all identified reports, the search might not have yielded a complete list, and communication with authors is not always productive [27,110]. As the proportions of *gyrB*, and *parC* mutants, and strains with PMQR genes were generally low, a missed study/data may not appreciably alter the respective pooled estimates. Nevertheless, we performed a *post hoc* sensitivity analysis to evaluate the potential effect of a missing study/data on the pooled proportion of *gyrA* mutant strains using the highest estimate as a proxy. As a result, the margin of equivalence was small (0.9%); the proxy-based estimate lies within the 95% confidence bounds of the estimate, and the estimates with and without a substitute did not significantly differ (Yates corrected X^2^ = 1.52, P = 0.217). Third, as ciprofloxacin was the FQ used to assess the phenotype and describe the genotypes, the genetic basis of potential cross-resistance to other FQs was not explored. Fourth, data on drug efflux and influx associated mechanisms are not available, and establishing a clear-cut relationship between genetic determinants and ciprofloxacin MICs is difficult.

We have considered studies with different designs [111], and established study validity by using the inclusion and exclusion criteria [27]. Several quality assessment tools (QATs)–checklists and scales–have been developed [111,112,113] for use in prevalence studies. Some of these tools have been recommended to assess the qualities of studies on infectious diseases [113,114]. However, the items and the weighting of the items are variable and inconsistent [112]. In addition, as critical components differ across domains and topics [21], we did not find a QAT matching the components addressed in this study. For this reason, a qualitative summary of the component quality items is presented (S2 Table and S3 Table). To supplement the summary, a *post hoc* sensitivity analysis on unmeasured confounding on the odds ratio of *gyrA* mutation in *S*. Typhi and NTS was performed. We considered a *gyrA* mutant prevalence of less than 10%; we approximated the RR from the OR estimate [115] and estimated the minimum bias factor and confounding strength as described by Mathur and VanderWeele (https://mmathur.shinyapps.io/meta_gui_2/), [116]. The input parameters and the output are given in S1 Fig, and the potential impact of bias is discussed here below. Our assessment is simplistic and intended to not run the risks of understating/overstating the bias and the evidence. Overall, other than the limitations described under the section on limitations (limited country representation and country level data), we presume that selection, reporting and confounding bias may not have a significant impact on the evidence on the preponderance of FQ-resistance determinants and the pooled estimates. Our assumptions are based on the following premises.

First, a cross-sectional sample of the patient population is the ideal sampling population to study low prevalence/rare conditions [117], and under such a setting 'random sampling' in its strictest sense is unethical, impractical and has not been employed. Recruitment and samplings were syndrome based (fever, gasteroeneritis and systemic infections). Most isolates have been recovered from samples collected in surveillance and cross-sectional studies, and the rest were isolated from samples collected in different populations, locations and over time. Assuming a crude infection prevalence of 1.8%, and a ciprofloxacin non-susceptible strain proportion of 6.2% (Table 3), the recovery rate of a non-susceptible strain is approximately one in 1000 samples. Furthermore, with the exception of isolates that failed to grow or lost, and serotype/phenotype based selections in a few cases–perhaps due to logistical reasons and study objectives–most isolates have been characterized. This study has captured data on serotypes most frequently implicated in invasive salmonellosis–widely reported (*S*. Typhi, *S*. Typhimurium, and *S*. Enteritidis) [1, 3, 4, 10, 28] and of local/regional importance (*S*. Concord, *S*. Isangi, *S*. Bovismorbificans, *S*. Stanleyville and *S*. Dublin) [1,10]. As these isolates were recovered from spatially and temporally divergent samples, it is reasonable to assume that they are close representatives of the predominant strains circulating in Africa. Consequently, the risk due to non-representation of the major serotypes and distortion of the estimates appear minimal.

Second, most of the data were from articles published in high-quality journals. However, determination of reporting bias is difficult [118,119,120], and the tools commonly employed to detect publication bias are not rigorous [120]. In this study we have captured data from studies with large and small sample sizes. Despite missed generic and phenotype/genotype information in some studies (S1 Table), genotype data was secured from most studies and for at least 94% of the isolates included herein. Systematic differences between unreported and reported data [118, 120] were not observed, and in each analysis only studies with complete outcomes [118] were considered.

Third, salmonellosis is principally a food-related infection largely of community setting origin [18]. Although there have been sporadic reports of health-care related *Salmonella* outbreaks [121], health-care associated infections in Africa [122] have been linked with Gram-negative bacilli other than *Salmonella*. Moreover, in a review of surveillance data of 33188 paediatric admissions in Kenya (2002–2009), Gram-negative bacilli other than *Salmonella* were the major causes of hospital-acquired bacteraemeia [123]. In this study, the difference between the pooled proportions of typhoidal and iNTS infections of community setting origin was marginal (Table 3). However, factors that may influence the occurrence of *Salmonella* and thus the phenotypic/genotypic proportions–e.g. malaria and HIV status–[5–7] have not been reported in most studies used to estimate pooled proportions. Nevertheless, the sensitivity analysis on unmeasured confounding on the occurrence of *gyrA* mutation in typhoidal and non-typhoidal *Salmonella* suggests the relative robustness of the pooled estimates to unmeasured confounding (S1 Fig).

Strength of evidence is related with the qualities of individual studies, and the size, reliability and robustness of the combined data [124]. Although the GRADE methodology [125] has been recommended to rate the evidence in systematic reviews addressing infectious diseases [113,126], the applicability of the criteria in prevalence studies is unclear [113,126]. Instead, we grouped studies as low risk studies to estimate pooled proportions, and low risk studies to calculate frequencies (S3 Table). To put the evidence in viewpoint, we considered the variance estimates/credibility intervals and the agreement between the meta- and frequency analyses (Tables 1–5). Accordingly, we judged the preponderance of genotypes (*gyrA* mutation predominance) as a first order (‘high quality’) evidence, and the pooled estimates as second rank (‘moderate quality’) evidence–subject to change depending on data accretion and over time.

### Implications and significance

FQ-resistant *Salmonella* is in the high tier of the global pathogen priority list for research and development of new antimicrobials [127]. The synopsis may be of importance to health-care providers and researchers in the domain. First, the summary indicates the likely distribution of FQ-resistant serotypes across regions and the range of resistance to FQs. Accordingly, given the socio-economic and agro-ecologic similarities of most countries in SSA, the data could be extrapolated to countries where sufficient data is not available. Second, it shows the landscape of research on FQ-resistance determinants in *Salmonella* isolates of Africa and the gaps to address in further research undertakings. Third, as the contribution of each FQ-resistance genetic determinant is indistinct, a global meta-analysis or large-scale testing of diverse collections of FQ-resistant isolates to known genetic determinants may provide explanation on the relative contributions of each determinant and offer clues as to whether additional mechanisms are operating or not. Fourth, the occurrence of *gyrA* mutations in a significant proportion of FQ-non-susceptible strains implies the potential development and utility of a mismatch-amplification mutation-assay [128] to rapidly detect FQ-resistant invasive strains. Fifth, the unevenness of estimates on the incidence of typhoidal and iNTS disease necessitates an update of the estimates using current data.

## Conclusions

Mutations at *gyrA* appear to account for ciprofloxacin resistance in most clinical strains. Compared to iNTS, estimates of *gyrA* mutant typhoidal *Salmonella* are heterogeneous. The composite data could be harnessed to develop a molecular assay that enables a rapid detection of FQ-resistant invasive strains. Further studies are needed to describe (i) the relative contributions of genetic determinants to FQ-resistance, (ii) the diversities of determinants, and the serotypes particularly in countries lacking adequate data, and (iii) on the incidence of typhoidal and iNTS disease.

## Supporting information

S1 Checklist PRISMA checklist. (DOC)

S1 Fig Sensitivity analysis on unmeasured confounding. (TIF)

S1 Table Characteristics of included studies. (PDF)

S2 Table Methodological qualities of included studies. (PDF)

S3 Table Assessment of bias. (PDF)

S4 Table Data used for meta-analyses. (PDF)
