# Supplementary figures and images for: Molecular epidemiology of fluoroquinolone resistant Salmonella in Africa: A systematic review and meta-analysis
Source: PLoS One. 2018 Feb 12;13(2):e0192575. doi: 10.1371/journal.pone.0192575 (PMC5809059; doi:10.1371/journal.pone.0192575)

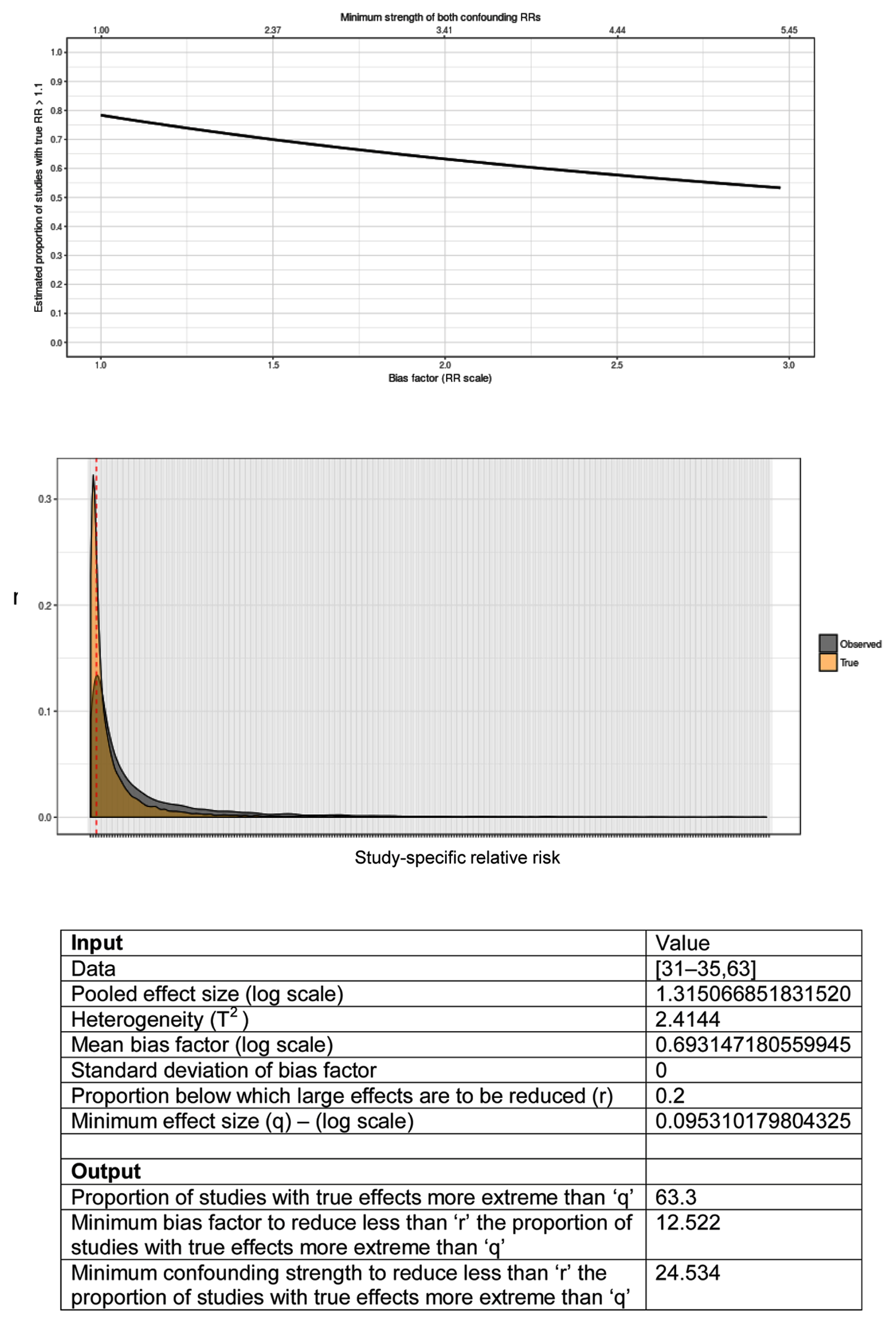

Supplement: S1 Fig — (TIF) [file pone.0192575.s002.tif]
