# Supplementary material for: Molecular epidemiology of fluoroquinolone resistant Salmonella in Africa: A systematic review and meta-analysis
Source: PLoS One. 2018 Feb 12;13(2):e0192575. doi: 10.1371/journal.pone.0192575 (PMC5809059; doi:10.1371/journal.pone.0192575)
Supplement: S1 Table — (PDF) [file pone.0192575.s003.pdf]

S1A Table. Included									
Reference	Year of collection	Country	Target population	Number of samples	Salmonella Group	Number DST_p	Number MDR	Number Nal^{ns}	Number Cip^{ns}
[31][a,†,@]	2011-4	DRC	Human	14110	TyS, NTS	940	721	nd	77
[32] [†,@]	2007-11	DRC	Human	9634	NTS	233	188	10	10
[33] [b,†]	2008-12	DRC	Human	626	TyS, NTS	103	97	1	1
[34][c,†,@]	2007-12	Ghana	Human	4694	TyS, NTS	261	169	nd	13
[35] [†]	2007-11	DRC	Human	9634	TyS	201	61	31	31
[36][†]	2011-3	Tanzania	Human	680	TyS, NTS	9	8	nd	0
[36][d,†]	2011-3	Kenya	Human	1251	TyS, NTS	48	38	nd	11
[36][d,†]	2011-3	MG	Human	2477	TyS, NTS	7	0	nd	0
[36][d,†]	2011-3	BF	Human	1674	TyS, NTS	15	0	nd	0
[36] [†]	2011-3	GB	Human	1021	TyS, NTS	3	0	nd	0
[36] [†]	2011-3	Senegal	Human	1058	TyS, NTS	9	0	nd	1
[37][e]	2001-8	Kenya	Human	2842	TyS	136	82	25	25
[38][f]	2008-15	Nigeria	Human	10133	TyS, NTS	128	68	7	7
[39][g]	2008-13	Mc	Human	Col	TyS	344	na	na	na
[40][h]	2009-11	Nigeria	H,A	1288	NTS	95	-	3	6
[41][i]	2000-2	Kenya	Human	nr(ap6000)	TyS	102	-	0	48
[42]	2003-7	SA	Human	nr(ap30000)	TyS	510	-	27	27
[43][j]	2010-2	Zambia	Human	nr(ap5529)	TyS	94	-	3	4
[44][k]	2001-6	Ethiopia	Human	54	NTS	36	-	3	3
[45][l]	2003-7	Ethiopia	Human	3419	NTS	35	-	nr	5
[46][m]	2004-9	Ethiopia	Human	655	NTS	26	-	4	4
[47][n]	2001	SA	Human	nr, Col	NTS	29	-	22	20
[48]	2012	Zambia	Human	2	NTS	2	-	2	2
[49]	2010	Ghana	Human	1	NTS	1	-	nd	1
[50][o]	2001-8	Mali	Human	61	NTS	38	-	4	nr
[51][p]	2000-9	Se, Ma	Human	nr(ap474)	NTS	9	-	5	6

[52][q,@]	2013-4	Ethiopia	H,A	3171	NTS	152	-	18	27
[53][r]	2012	Libya	Human	1	NTS	1	-	nd	1
[54][s]	2003-11	Mr, NA	Human	nr, Col	NTS	30,6	-	nr,5	19,5
[55]	2005-13	Mc	H,A/p	Col	NTS	9	-	6	8
[56][t,@]	2000-8	Mc	H,A/p	Col	NTS	45	-	40	40
[57][u]	nr	Egypt	Ap	1600	NTS	69	-	29	19
[58][v]	2006-7	Egypt	Animal	220	NTS	9	-	4	3
[59][w]	2008-11	Tunisia	AP	nr(ap9296)	NTS	377	-	110	104
[60][x]	2002-5	Morocco	AP	8522	NTS	80	-	1	nd
[61] [y]	2002-9	Tunisia	AP	nr(ap1367)	NTS	37	-	26	nd
[62][z]	2010-1	Nigeria	Animal	306	NTS	224	-	34	16
[63]	2011-4	DRC	Human	nr(ap5944)	TyS/NTS	107	85	2	2
[64]	2015	DRC	Human	1	TyS	1	-	1	1
[65]	2007	Eg, Et	Human	4	NTS	4	-	0	4
[66]	2010-2	Chad	Human	420	NTS	43	-	2	2
[67]	1997-04	Malawi	Human	Col	NTS	14	-	0	0

A, animal; ap, approximated; AP, animal product; A/P, animal or animal product; BF, Burkina-Faso; CEWSN, Central, Eastern, Western, Southern and Northern Africa; Cip^{ns}, ciprofloxacin non-susceptible; Col, collection; DRC, Democratic Republic of Congo; DST$_{p,}$ drug sensitivity testing performed; Eg, Egypt; Et, Ethiopia; GB, Guinea-Bissau; H, human; MC, multi-country; MDR, multi-drug resistant; MG, Madagaskar; Mr, Morocco; na, not applicable; NA, North African countries; -, not considered; Nal^{ns}, nalidixic acid non-susceptible; nd, not done; nr, not reported; NTS, non-typhoidal *Salmonella*; Ref, references; SA, South Africa; Se, Senegal; TyS, typhoidal *Salmonella*.

[†]Data were used to estimate prevalence of invasive salmonellosis and *gyA* mutant infection. DST and gene detection have not been performed in all [36, 38] or a proportion [31, 33] of NTS isolates.

[@]Online/supplementary information was obtained.

[a]Numbers of samples and numbers of isolates were extracted from Figure 2 and Table 3. The number of BSI episodes was considered as the number of samples.

[b]The data from 2008 to October 2011 was retrospective and actual number of samples was not given. Enhanced sampling was done after November 2011 and isolates were recovered from children.

[c]Number of MDR strains was calculated (MDR = 108 + 59 + 2 = 169).
[d]Missing *S*. Typhi strains were subtracted – Kenya (6), Burkina-Faso (3), and Madagascar (1).
[e]Sample size (n = 0.42*6750) and number of MDR isolates (MDR = 0.78*105 = 82) were calculated. The number of Cip^{ns} strains was adjusted: 9 strains had the following characteristics: Nal MIC = 8-16µg/mL, Cip MIC = 0.02-0.1µg/mL, and Ser83→Leu substitution.
[f]Samples were collected from 2008 to 2015. The phenotype/genotype data is for isolates collected between 2008 and 2013. The numbers of MDR, and Cip^{ns} strains were extrapolated from the genotype data (MDR = *cat* + bla_{TEM} + *dfrA*-sul1 = chloramphenicol + ampicillin + cotrimoxazole; *qnrS*/*gyrA* = quinolone non-susceptible).
[g]Number of isolates was extracted from countries listed in supplementary Table 1. The data on *parC* and *parE* were extrapolated from supplementary Figure 4.
[h]Samples, and isolates recovered from rodents, lizards, vegetables, and fish intestines were excluded. Isolates with reduced susceptibility to ciprofloxacin have been subjected to gene detection.
[i]Isolates were collected over two years, from different locations, with no clear source, and had variable drug resistance profiles.
[j]Isolates were collected over two years, from different locations, and had variable drug resistance profiles. Sample size was approximated.
[k]Sample size = adoptees in France (28) + adoptees in Norway (26) = 54
[l] Number of isolates (n) was calculated, n = 5/0.14.
[m]Three *gyrA* mutants and one *qnr* positive strain were considered.
[n]Twenty-six isolates (CIP MICs ≥ 0.125µg/mL) were considered as quinolone non-susceptible.
[o]Three isolates from mothers were considered as duplicates, and were excluded.
[p]Number of isolates collected between 2000-2008 was1478. The number of Nal^{ns} strains was 57. Only ten ESBLs positive isolates were subjected to genotype detection. Sample size was approximated for ten ESBL isolates only. The number of Nal^{ns} and Cip^{ns} strains was adjusted.
[q]Sample size was extrapolated from the thesis.
[r]Isolates had similar drug resistance phenotypes, and only one was considered.
[s]Numbers of Nal^{ns} strains in Morocco was not reported.
[t]Number of Cip^{ns} strains was adjusted.
[u]Data was extracted (tallied) from the table on AMR profiles.
[v]One of the strains was non-susceptible to norfloxacin.
[w]Isolates from clams were excluded. Sample size for chicken (251/0.027) was calculated using proportion from Tunisia (18/665) [61].

[x]Isolates from mollusks, spice, pastry and water were excluded; sample size was taken from Bouchrif et al. J Infect Dev Ctries. 2009; 3(1):35-40.<br>[y]Sample size was calculated [(37/18)*665].<br>[z]Fourteen isolates were examined to detect PMQR genes.

S1B Table. Not included		
Reference		Reason
Diop et al.	PLoS ONE. 2016.	Unclear mutant data.
Saleh et al.	J Infect Dev Ctries. 2014.	Incomplete *gyrA* data.
Moawad et al.	Gut Pathog. 2017.	Unclear serotype data.
Sambe-ba et al.	J Glob Antimicrob Resist. 2013	Letter
Keddy et al.	Emerg Infect Dis. 2010.	Letter
Bouchrif et al.	Méd Mal Infect. 2008.	Letter
Weill et al.	Emerg Infect Dis.2006.	Letter
Govender et al.	J Med Microbiol. 2009.	Correspondence
Phoba et al.	Emerg Infect Dis. 2012.	Letter
Ktari et al.	J Antimicrob Chemother. 2015.	Letter
