# Supplementary material for: Molecular epidemiology of fluoroquinolone resistant Salmonella in Africa: A systematic review and meta-analysis
Source: PLoS One. 2018 Feb 12;13(2):e0192575. doi: 10.1371/journal.pone.0192575 (PMC5809059; doi:10.1371/journal.pone.0192575)
Supplement: S2 Table — (PDF) [file pone.0192575.s004.pdf]

Ref.	Study design	Target population	Sampling population†	H–age group	Sample	Sampling	DST_m	Cip^s 0.06µg/mL	GD
[31]	Srv-cs-mc	Human	Cm	All	Blood	Pr	MIC	≤	PCR/S
[32]	Srv-cs-mc	Human	Cm	All	Blood	Pr	MIC	≤	PCR/S
[33]	Srv-cs-sc	Human	Cm	All	Blood	Pr	MIC	≤	PCR/S
[34]	Srv-cs-sc	Human	Cm	Chld	Blood	Pr	MIC	≤	PCR/S
[35]	Srv-cs-mc	Human	Cm	All	Blood	Pr	MIC	≤	PCR/S
[36]	Srv-cs-sc	Human	Cm	All	Blood	Pr	MIC	≤	PCR/S
[36]	Srv-cs-sc	Human	Cm	All	Blood	Pr	MIC	≤	PCR/S
[36]	Srv-cs-mc	Human	Cm	All	Blood	Pr	MIC	≤	PCR/S
[36]	Srv-cs-mc	Human	Cm	All	Blood	Pr	MIC	≤	PCR/S
[36]	Srv-cs-sc	Human	Cm	All	Blood	Pr	MIC	≤	PCR/S
[36]	Srv-cs-sc	Human	Cm	All	Blood	Pr	MIC	≤	PCR/S
[37]	Srv-cs-mc	Human	Cm	All	Blood	Pr	MIC	$\leq_a$	PCR/S
[38][a]	Srv-cs-mc	Human	Cm	Chld	Blood	Pr	MIC	Gn	WGS
[39][b]	MC-cl	Human	nr	nr	Blood	Rt	Gn	Gn	WGS
[40]	Crs-mc	H, C, Cl, P	Cm,Sl,Fr	Adl	St, Mx	Pr	MIC	≤	PCR
[41]	Hl-mc-cl	Human	Cm	Adl	Blood	Un	MIC	>	PCR/S
[42]	Srv-lb-mc	Human	Cm	nr	nr	Rt	MIC	>	PCR/S
[43]	Hl-sc-cl	Human	Cm	All	Bl,St	Rt	MIC	≤	WGS
[44]	MC-cl	Human	Ad	Chld	Stool	Rt	nr	nr	PCR
[45]	MC-cl	Human	Ad	Chld	Stool	Rt	MIC	>	PCR/S
[46]	Lb-cl	Human	Ad	Chld	Stool	Rt	ZD	na	PCR/S
[47]	Hl-sc-cl	Human	Cm	Chld	Stool	Rt	ZD	na	PCR/S
[48]	Cas-re	Human	Cs	Adl	Stool	na	MIC	≤	WGS
[49]	Cas-re	Human	Cs	17y	Blood	na	MIC	≤	PCR/S
[50]	Srv-cs-sc	Human	Ad	Chld	Stool	Pr	ZD	na	PCR/S
[51]	Srv-lb-mc	Human	Cm	nr	Bl, St	Rt	MIC	$\leq_a$	PCR/S
[52]	Crs-mc	H,C,Sw,P	Cm,Fr	All	St, Fc	Pr	ZD	na	PCR/S

[53]	Cas-re	Human	Cs	Adl	As	na	MIC	>	PCR/S
[54]	MC-cl	Human	Cm,Tr	nr	nr	Rt	MIC	>	PCR/S
[55]	MC-cl	H,A/P(Sw,P)	Tr,nr	nr	nr	Rt	MIC	>	PCR/S
[56]	MC-cl	H, A/P(P)	Tr,nr	nr	nr	Rt	MIC	>	PCR/S
[57]	Un-mc	AP(C,P)	Mk	na	Mt, Da	Pr	ZD	na	PCR
[58]	Crs-mc	A(C)	Fr	na	Feces	Pr	ZD	na	PCR
[59]	Srv-lb-mc	AP(R?,P)	nr	na	Meat	Rt	MIC	>	PCR
[60]	Un-mc	AP (R?, P)	Sl,Ho	na	Mixed	Pr	nr	nr	PCR/S
[61]	Crs/cl-mc	AP (P)	Sl	na	Meat	Pr /Rt	ZD	na	PCR
[62]	Crs-mc	A (Sw)	Fr	na	Feces	Pr	ZD	na	PCR
[63]	Srv-cs-mc	Human	Cm	All	Blood	Rt	MIC	≤	PCR/S
[64]	Cas-re	Human	Cs	Chld	Blood	na	MIC	≤	PCR/S
[65]	Un-cl	Human	Ad,Tr	All	nr	na	MIC	>	PCR/S
[66]	Crs-mc	Human	Cm	All	St	Pr	ZD	na	PCR
[67]	Hl-sc-cl	Human	Cm	All	Blood	Rt	ZD	na	WGS

A, animal; Ad, adoptees; Adl, adults; Ap, animal product; A/P, animal or animal product; As, Anal swab; Bl, blood; BF, Burkina-Faso; C, cattle; Cas-re, case report; Cip^s, ciprofloxacin susceptibility; Chld, children; Cl, camel; Cm, community; Cs, case; Crs-mc, cross-sectional multicenter; Crs/cl-mc, cross-sectional/collections multicenter; Da, dairy products; DRC, Democratic Republic of Congo; DST_m, drug sensitivity test measurement; Eg, Egypt; Et, Ethiopia; Fc, feces; Fr, farms; GB, Guinea-Bissau; GD, gene detection method; Gn, genotype based; Ho, hotels and others; H, human; Hl-mc-cl, hospital multicenter collection; Hl-sc-cl, hospital single center collection; $\leq_a$, adjusted susceptibility break-point level; Lb-cl, laboratory collection; Ma, Mali; MC, multi-country; MC-cl, Multi-country collection; MIC, minimum inhibitory concentration; MG, Madagaskar; Mk, market; Mr, Morocco; Mt, meat; Mx, mixed samples; NA, North African countries; na, not applicable; nr, not reported; Pr, prospective sampling; PCR/S, Polymerase Chain Reaction and sequencing; P, poultry; R?, ruminant species not identified; Rt, retrospective samples; Se, Senegal; Sl, slaughterhouse; Srv-cs-mc, surveillance case-series and multi-center; Srv-cs-sc, surveillance case-series and single center; Srv-lb-mc, surveillance laboratory-based and multi-center; St, stool; Sw, swine; Tr, travel associated; Un-mc, Unclear multicenter; WGS, whole genome sequence based; ZD, zone diameter – disk diffusion test.
[a]MIC was determined but not reported.

[b]Most *S*. Typhi were from blood samples. The data were used to estimate pooled proportion of *parC* mutants.
‡ *Salmonella* is largely of community origin [18]. If onset was not reported, infection was considered as community-acquired.
