# Supplementary material for: Molecular epidemiology of fluoroquinolone resistant Salmonella in Africa: A systematic review and meta-analysis
Source: PLoS One. 2018 Feb 12;13(2):e0192575. doi: 10.1371/journal.pone.0192575 (PMC5809059; doi:10.1371/journal.pone.0192575)
Supplement: S3 Table — (PDF) [file pone.0192575.s005.pdf]

S3A Table. Quality/risk of bias assessment tool.						
JBI	M	Quality items	Yes	No	Un	Na
Q1	Q1	Was the target population described?				
Q4	Q2	Was the sampling population described?				
Q3	Q3	Was the sample size > 384				
Q2	Q4	Was the sampling prospective?				
	Q5	Was the sample/specimen specified?				
	Q6	Was the sample/specimen blood?				
Q6	Q7	Was ciprofloxacin susceptibility MIC ≤ 0.06 µg/mL?				
	Q8	Were all quinolone non-susceptible strains genotyped?				
Q7	Q9	Was genotype detection phenotype-PCR/sequence based?				
M, modified; Un, unclear; Na, not applicable.<br>Modified from Joanna Briggs Institute (JBI) [28]. Quality items 5, 8 and 9 in JBI were handled in Q3.						

S3B Table. Relative risks of bias of inclusion of studies in the meta- and frequency analyses.										
Quality items									Low risk = Yes	
Ref.[‡]	Q1	Q2	Q3	Q4	Q5	Q6	Q7	Q8	Q9	Meta-	Frequency
[31]	Yes	Yes	Yes	Yes	Yes	Yes	Yes	Yes	Yes	Yes	Yes
[32]	Yes	Yes	Yes	Yes	Yes	Yes	Yes	Yes	Yes	Yes	Yes
[33]	Yes	Yes	Yes	Yes	Yes	Yes	Yes	Yes	Yes	Yes	Yes
[34]	Yes	Yes	Yes	Yes	Yes	Yes	Yes	Yes	Yes	Yes	Yes
[35]	Yes	Yes	Yes	Yes	Yes	Yes	Yes	Yes	Yes	Yes	Yes
[36]	Yes	Yes	Yes	Yes	Yes	Yes	Yes	Yes	Yes	Yes	Yes
[36]	Yes	Yes	Yes	Yes	Yes	Yes	Yes	Yes	Yes	Yes	Yes
[36]	Yes	Yes	Yes	Yes	Yes	Yes	Yes	Yes	Yes	Yes	Yes
[36]	Yes	Yes	Yes	Yes	Yes	Yes	Yes	Yes	Yes	Yes	Yes
[36]	Yes	Yes	Yes	Yes	Yes	Yes	Yes	Yes	Yes	Yes	Yes
[36]	Yes	Yes	Yes	Yes	Yes	Yes	Yes	Yes	Yes	Yes	Yes
[37]	Yes	Yes	Yes	Yes	Yes	Yes	Yes	Yes	Yes	Yes	Yes
[38]	Yes	Yes	Yes	Yes	Yes	Yes	Yes	Yes	No	Yes	Yes
[39]	Yes	No	Yes	No	Yes	Yes	na	na	No	Yes	Yes
[63]	Yes	Yes	Yes	No	Yes	Yes	Yes	Yes	Yes	Yes	Yes
[40]	Yes	Yes	Yes	Yes	Yes	No	Yes	No	Yes	No	Yes
[41]	Yes	Yes	Yes	Un	Yes	Yes	No	No	Yes	No	Yes
[42]	Yes	Yes	Yes	No	No	Un	No	No	Yes	No	Yes
[43]	Yes	Yes	Yes	No	Yes	Yes	Yes	No	No	No	Yes

[44]	Yes	Yes	No	No	Yes	No	No	Yes	Yes	No	Yes
[45]	Yes	Yes	Yes	No	Yes	No	No	Yes	Yes	No	Yes
[46]	Yes	Yes	Yes	No	Yes	No	No	Yes	Yes	No	Yes
[47]	Yes	Yes	Yes	No	Yes	No	No	Yes	Yes	No	Yes
[48]	Yes	Yes	No	na	Yes	No	Yes	na	No	No	Yes
[49]	Yes	Yes	No	na	Yes	No	Yes	na	Yes	No	Yes
[50]	Yes	Yes	No	Yes	Yes	No	No	Yes	Yes	No	Yes
[51]	Yes	Yes	Yes	No	Yes	No	Yes	No	Yes	No	Yes
[52]	Yes	Yes	Yes	Yes	Yes	No	No	No	Yes	No	Yes
[53]	Yes	Yes	na	na	Yes	No	Yes	Yes	Yes	No	Yes
[54]	Yes	Yes	na	No	No	na	No	No	Yes	No	Yes
[55]	Yes	Y/N	na	No	No	na	No	Yes	Yes	No	Yes
[56]	Yes	Y/N	na	No	No	na	No	Yes	Yes	No	Yes
[57]	Yes	Yes	Yes	Yes	Yes	No	No	Yes	Yes	No	Yes
[58]	Yes	Yes	No	Yes	Yes	No	No	Yes	Yes	No	Yes
[59]	Yes	Yes	Yes	Yes	Yes	No	No	Yes	Yes	No	Yes
[60]	Yes	Yes	Yes	Yes	Yes	No	No	Yes	Yes	No	Yes
[61]	Yes	Yes	Yes	Y/N	Yes	No	No	Yes	Yes	No	Yes
[62]	Yes	Yes	No	Yes	Yes	No	No	No	Yes	No	Yes
[64]	Yes	Yes	na	na	Yes	Yes	Yes	Yes	Yes	No	Yes
[65]	Yes	Yes	na	na	No	Un	No	Yes	Yes	No	Yes
[66]	Yes	Yes	Yes	Yes	Yes	No	No	No	No	No	Yes
[67]	Yes	Yes	na	No	Yes	Yes	na	na	No	No	Yes

Na, not applicable; Un, unclear; Y/N, yes for some and no for others.

‡ Studies shaded in green are relatively homogeneous with respect to the sampling population and methods, and data were used in meta- and frequency analyses. Studies shaded in yellow are relatively heterogeneous with respect to the sampling population and methods, and data were used to calculate relative and cumulative frequencies (gene mutations, mutation positions, substituted amino acids, PMQR genes and mutant serotypes).
