# Supplementary material for: Molecular epidemiology of fluoroquinolone resistant Salmonella in Africa: A systematic review and meta-analysis
Source: PLoS One. 2018 Feb 12;13(2):e0192575. doi: 10.1371/journal.pone.0192575 (PMC5809059; doi:10.1371/journal.pone.0192575)
Supplement: S4 Table — (PDF) [file pone.0192575.s006.pdf]

Ref.	Phenotype/ Genotype	Serotype	N	n	%	$t = \sin^{-1}(\sqrt{n/N+1}) + \sin^{-1}(\sqrt{n+1/N+1})$	Se t $=\sqrt{1/N+0.5}$	$P = 0.5\{1\text{-sgn} (\cos t) [1- (N (\sin t)^2 + (\sin t)^2 -1/(N \sin t))^2]^{0.5}\}$
[31]	MDR	*S.* Typhimurium	386	348	90.2	2.499829	0.050866	0.901554
[32]	MDR	*S.* Typhimurium	184	160	87.0	2.396697	0.073621	0.869565
[33]	MDR	*S.* Typhimurium	56	55	98.2	2.820319	0.133038	0.982143
[34]	MDR	*S.* Typhimurium	129	108	83.7	2.304018	0.087875	0.837209
[63]	MDR	*S.* Typhimurium	45	40	88.9	2.436108	0.14825	0.888889
[31]	MDR	*S.* Enteritidis	390	311	79.7	2.206015	0.050605	0.797436
[32]	MDR	*S.* Enteritidis	42	25	59.5	1.757986	0.153393	0.595238
[33]	MDR	*S.* Enteritidis	29	28	96.6	2.696831	0.184115	0.965517
[34]	MDR	*S.* Enteritidis	19	2	10.5	0.71945	0.226455	0.105263
[63]	MDR	*S.* Enteritidis	50	41	82.0	2.249327	0.14072	0.82
[31]	MDR	*S.* Typhi	164	62	37.8	1.325928	0.077968	0.378049
[33]	MDR	*S.* Typhi	18	6	33.3	1.248994	0.232495	0.333333
[34]	MDR	*S.* Typhi	89	59	66.3	1.898896	0.105703	0.662921
[35]	MDR	*S.* Typhi	201	61	30.3	1.168976	0.070447	0.303483
[36]	MDR	*S.* Typhi	48	38	79.2	2.179257	0.143592	0.791667
[36][‡]	MDR	*S.* Typhi	16	0	0.0	0.244979	0.246183	0
[36]	MDR	*S.* Typhi	15	0	0.0	0.25268	0.254	0
[36][‡]	MDR	*S.* Typhi	10	0	0.0	0.306277	0.308607	0
[37]	MDR	*S.* Typhi	136	82	60.3	1.776632	0.085592	0.602941
[38]	MDR	*S.* Typhi	128	68	53.1	1.632854	0.088216	0.53125
[63]	MDR	*S.* Typhi	11	3	27.3	1.139078	0.294884	0.272727
[31]	Cip^{ns}	*S.* Typhimurium	386	8	2.1	0.297373	0.050866	0.020725
[32]	Cip^{ns}	*S.* Typhimurium	184	9	4.9	0.457036	0.073621	0.048913
[33]	Cip^{ns}	*S.* Typhimurium	56	1	1.8	0.321274	0.133038	0.017857
[34]	Cip^{ns}	*S.* Typhimurium	129	3	2.3	0.328825	0.087875	0.023256

[63]	Cip^{ns}	*S.* Typhimurium	45	1	2.2	0.358037	0.14825	0.022222
[31]	Cip^{ns}	*S.* Enteritidis	390	7	1.8	0.277736	0.050605	0.017949
[32]	Cip^{ns}	*S.* Enteritidis	42	0	0.0	0.153096	0.153393	0
[33]	Cip^{ns}	*S.* Enteritidis	29	0	0.0	0.183604	0.184115	0
[34]	Cip^{ns}	*S.* Enteritidis	19	10	52.6	1.62088	0.226455	0.526316
[63]	Cip^{ns}	*S.* Enteritidis	50	0	0.0	0.14049	0.14072	0
[31]	Cip^{ns}	*S.* Typhi	164	61	37.2	1.313414	0.077968	0.371951
[33]	Cip^{ns}	*S.* Typhi	18	0	0.0	0.231477	0.232495	0
[34]	Cip^{ns}	*S.* Typhi	89	0	0.0	0.105605	0.105703	0
[35]	Cip^{ns}	*S.* Typhi	201	31	15.4	0.811881	0.070447	0.154229
[36]	Cip^{ns}	*S.* Typhi	48	11	22.9	1.011292	0.143592	0.229167
[36]‡	Cip^{ns}	*S.* Typhi	16	0	0.0	0.244979	0.246183	0
[36]	Cip^{ns}	*S.* Typhi	15	0	0.0	0.25268	0.254	0
[36]‡	Cip^{ns}	*S.* Typhi	10	0	0.0	0.306277	0.308607	0
[37]	Cip^{ns}	*S.* Typhi	136	25	18.4	0.892118	0.085592	0.183824
[38]	Cip^{ns}	*S.* Typhi	128	7	5.5	0.486783	0.088216	0.054688
[63]	Cip^{ns}	*S.* Typhi	11	1	9.1	0.713377	0.294884	0.090909
[31]	MDR-Cip^{ns}	*S.* Typhimurium	386	7	1.8	0.279177	0.050866	0.018135
[32]	MDR-Cip^{ns}	*S.* Typhimurium	184	8	4.3	0.431872	0.073621	0.043478
[33]	MDR-Cip^{ns}	*S.* Typhimurium	56	1	1.8	0.321274	0.133038	0.017857
[34]	MDR-Cip^{ns}	*S.* Typhimurium	129	3	2.3	0.328825	0.087875	0.023256
[63]	MDR-Cip^{ns}	*S.* Typhimurium	45	1	2.2	0.358037	0.14825	0.022222
[31]	MDR-Cip^{ns}	*S.* Enteritidis	390	2	0.5	0.159287	0.050605	0.005128
[32]	MDR-Cip^{ns}	*S.* Enteritidis	42	0	0.0	0.153096	0.153393	0
[33]	MDR-Cip^{ns}	*S.* Enteritidis	29	0	0.0	0.183604	0.184115	0
[34]	MDR-Cip^{ns}	*S.* Enteritidis	19	2	10.5	0.71945	0.226455	0.105263
[63]	MDR-Cip^{ns}	*S.* Enteritidis	50	0	0.0	0.14049	0.14072	0
[31]	MDR-Cip^{ns}	*S.* Typhi	164	30	18.3	0.888828	0.077968	0.182927
[33]	MDR-Cip^{ns}	*S.* Typhi	18	0	0.0	0.231477	0.232495	0

[34]	MDR-Cip^{ns}	*S*. Typhi	89	0	0.0	0.105605	0.105703	0
[35]	MDR-Cip^{ns}	*S*. Typhi	201	15	7.5	0.561286	0.070447	0.074627
[36]	MDR-Cip^{ns}	*S*. Typhi	48	8	16.7	0.858863	0.143592	0.166667
[36]‡	MDR-Cip^{ns}	*S*. Typhi	16	0	0.0	0.244979	0.246183	0
[36]	MDR-Cip^{ns}	*S*. Typhi	15	0	0.0	0.25268	0.254	0
[36]‡	MDR-Cip^{ns}	*S*. Typhi	10	0	0.0	0.306277	0.308607	0
[37]	MDR-Cip^{ns}	*S*. Typhi	136	25	18.4	0.892118	0.085592	0.183824
[38]	MDR-Cip^{ns}	*S*. Typhi	128	3	2.3	0.330109	0.088216	0.023438
[63]	MDR-Cip^{ns}	*S*. Typhi	11	0	0.0	0.292843	0.294884	0
[31]	nMDR-Cip^{ns}	*S*. Typhimurium	386	1	0.3	0.122805	0.050866	0.002591
[32]	nMDR-Cip^{ns}	*S*. Typhimurium	184	1	0.5	0.177751	0.073621	0.005435
[33]	nMDR-Cip^{ns}	*S*. Typhimurium	56	0	0.0	0.132844	0.133038	0
[34]	nMDR-Cip^{ns}	*S*. Typhimurium	129	0	0.0	0.087819	0.087875	0
[63]	nMDR-Cip^{ns}	*S*. Typhimurium	45	0	0.0	0.147981	0.14825	0
[31]	nMDR-Cip^{ns}	*S*. Enteritidis	390	5	1.3	0.23752	0.050605	0.012821
[32]	nMDR-Cip^{ns}	*S*. Enteritidis	42	0	0.0	0.153096	0.153393	0
[33]	nMDR-Cip^{ns}	*S*. Enteritidis	29	0	0.0	0.183604	0.184115	0
[34]	nMDR-Cip^{ns}	*S*. Enteritidis	19	8	42.1	1.420034	0.226455	0.421053
[63]	nMDR-Cip^{ns}	*S*. Enteritidis	50	0	0.0	0.14049	0.14072	0
[31]	nMDR-Cip^{ns}	*S*. Typhi	164	31	18.9	0.904345	0.077968	0.189024
[33]	nMDR-Cip^{ns}	*S*. Typhi	18	0	0.0	0.231477	0.232495	0
[34]	nMDR-Cip^{ns}	*S*. Typhi	89	0	0.0	0.105605	0.105703	0
[35]	nMDR-Cip^{ns}	*S*. Typhi	201	16	8.0	0.579625	0.070447	0.079602
[36]	nMDR-Cip^{ns}	*S*. Typhi	48	3	6.3	0.539785	0.143592	0.0625
[36]‡	nMDR-Cip^{ns}	*S*. Typhi	16	0	0.0	0.244979	0.246183	0
[36]	nMDR-Cip^{ns}	*S*. Typhi	15	0	0.0	0.25268	0.254	0
[36]‡	nMDR-Cip^{ns}	*S*. Typhi	10	0	0.0	0.306277	0.308607	0
[37]	nMDR-Cip^{ns}	*S*. Typhi	136	0	0.0	0.08554	0.085592	0
[38]	nMDR-Cip^{ns}	*S*. Typhi	128	4	3.1	0.375182	0.088216	0.03125

[63]	nMDR-Cip^{ns}	*S.* Typhi	11	1	9.1	0.713377	0.294884	0.090909
[31]	*gyrA*	*S.* Typhimurium	386	7	1.8	0.279177	0.050866	0.018135
[32]	*gyrA*	*S.* Typhimurium	184	9	4.9	0.457036	0.073621	0.048913
[33]	*gyrA*	*S.* Typhimurium	56	1	1.8	0.321274	0.133038	0.017857
[34]	*gyrA*	*S.* Typhimurium	129	2	1.6	0.276856	0.087875	0.015504
[63]	*gyrA*	*S.* Typhimurium	45	1	2.2	0.358037	0.14825	0.022222
[31]	*gyrA*	*S.* Enteritidis	390	7	1.8	0.277736	0.050605	0.017949
[32]	*gyrA*	*S.* Enteritidis	42	0	0.0	0.153096	0.153393	0
[33]	*gyrA*	*S.* Enteritidis	29	0	0.0	0.183604	0.184115	0
[34]	*gyrA*	*S.* Enteritidis	19	9	47.4	1.520713	0.226455	0.473684
[63]	*gyrA*	*S.* Enteritidis	50	0	0.0	0.14049	0.14072	0
[31]	*gyrA*	*S.* Typhi	164	55	33.5	1.237373	0.077968	0.335366
[33]	*gyrA*	*S.* Typhi	18	0	0.0	0.231477	0.232495	0
[34]	*gyrA*	*S.* Typhi	89	0	0.0	0.105605	0.105703	0
[35]	*gyrA*	*S.* Typhi	201	31	15.4	0.811881	0.070447	0.154229
[36]	*gyrA*	*S.* Typhi	48	8	16.7	0.858863	0.143592	0.166667
[36]‡	*gyrA*	*S.* Typhi	16	0	0.0	0.244979	0.246183	0
[36]	*gyrA*	*S.* Typhi	15	0	0.0	0.25268	0.254	0
[36]‡	*gyrA*	*S.* Typhi	10	0	0.0	0.306277	0.308607	0
[37]	*gyrA*	*S.* Typhi	136	25	18.4	0.892118	0.085592	0.183824
[38]	*gyrA*	*S.* Typhi	128	6	4.7	0.452479	0.088216	0.046875
[63]	*gyrA*	*S.* Typhi	11	1	9.1	0.713377	0.294884	0.090909
[31]	*gyrB*	*S.* Typhimurium	386	0	0.0	0.050855	0.050866	0
[32]	*gyrB*	*S.* Typhimurium	184	0	0.0	0.073588	0.073621	0
[33]	*gyrB*	*S.* Typhimurium	56	0	0.0	0.132844	0.133038	0
[34]	*gyrB*	*S.* Typhimurium	129	1	0.8	0.212174	0.087875	0.007752
[63]	*gyrB*	*S.* Typhimurium	45	0	0.0	0.147981	0.14825	0
[31]	*gyrB*	*S.* Enteritidis	390	0	0.0	0.050594	0.050605	0
[32]	*gyrB*	*S.* Enteritidis	42	0	0.0	0.153096	0.153393	0

[33]	*gyrB*	*S.* Enteritidis	29	0	0.0	0.183604	0.184115	0
[34]	*gyrB*	*S.* Enteritidis	19	0	0.0	0.225513	0.226455	0
[63]	*gyrB*	*S.* Enteritidis	50	0	0.0	0.14049	0.14072	0
[31]	*gyrB*	*S.* Typhi	164	0	0.0	0.077929	0.077968	0
[33]	*gyrB*	*S.* Typhi	18	0	0.0	0.231477	0.232495	0
[34]	*gyrB*	*S.* Typhi	89	0	0.0	0.105605	0.105703	0
[35]	*gyrB*	*S.* Typhi	201	0	0.0	0.070418	0.070447	0
[36]	*gyrB*	*S.* Typhi	48	3	6.3	0.539785	0.143592	0.0625
[36]‡	*gyrB*	*S.* Typhi	16	0	0.0	0.244979	0.246183	0
[36]	*gyrB*	*S.* Typhi	15	0	0.0	0.25268	0.254	0
[36]‡	*gyrB*	*S.* Typhi	10	0	0.0	0.306277	0.308607	0
[37]	*gyrB*	*S.* Typhi	136	0	0.0	0.08554	0.085592	0
[38]	*gyrB*	*S.* Typhi	128	0	0.0	0.088159	0.088216	0
[63]	*gyrB*	*S.* Typhi	11	0	0.0	0.292843	0.294884	0
[31]	*parC*	*S.* Typhimurium	386	0	0.0	0.050855	0.050866	0
[32]	*parC*	*S.* Typhimurium	184	0	0.0	0.073588	0.073621	0
[33]	*parC*	*S.* Typhimurium	56	0	0.0	0.132844	0.133038	0
[34]	*parC*	*S.* Typhimurium	129	0	0.0	0.087819	0.087875	0
[63]	*parC*	*S.* Typhimurium	45	0	0.0	0.147981	0.14825	0
[31]	*parC*	*S.* Enteritidis	390	0	0.0	0.050594	0.050605	0
[32]	*parC*	*S.* Enteritidis	42	0	0.0	0.153096	0.153393	0
[33]	*parC*	*S.* Enteritidis	29	0	0.0	0.183604	0.184115	0
[34]	*parC*	*S.* Enteritidis	19	0	0.0	0.225513	0.226455	0
[63]	*parC*	*S.* Enteritidis	50	0	0.0	0.14049	0.14072	0
[31]	*parC*	*S.* Typhi	164	0	0.0	0.077929	0.077968	0
[33]	*parC*	*S.* Typhi	18	0	0.0	0.231477	0.232495	0
[34]	*parC*	*S.* Typhi	89	0	0.0	0.105605	0.105703	0
[35]	*parC*	*S.* Typhi	201	0	0.0	0.070418	0.070447	0
[36]	*parC*	*S.* Typhi	48	0	0.0	0.143348	0.143592	0

[36]‡	*parC*	*S.* Typhi	16	0	0.0	0.244979	0.246183	0
[36]	*parC*	*S.* Typhi	15	0	0.0	0.25268	0.254	0
[36]‡	*parC*	*S.* Typhi	10	0	0.0	0.306277	0.308607	0
[37]	*parC*	*S.* Typhi	136	0	0.0	0.08554	0.085592	0
[38]	*parC*	*S.* Typhi	128	0	0.0	0.088159	0.088216	0
[39]	*parC*	*S.* Typhi	344	0	0.0	0.053864	0.053877	0
[63]	*parC*	*S.* Typhi	11	0	0.0	0.292843	0.294884	0
[31]	*qnr (A,B,S)*	NTS	776	1	0.1	0.086639	0.035886	0.001289
[32]	*qnr (A,B,S)*	NTS	226	0	0.0	0.066421	0.066446	0
[33]	*qnr (A,B,S)*	NTS	85	0	0.0	0.108043	0.108148	0
[34]	*qnr (A,B,S)*	NTS	148	0	0.0	0.082015	0.082061	0
[63]	*qnr (A,B,S)*	NTS	95	0	0.0	0.10224	0.102329	0
[31]	*qnr (A,B,S)*	TyS	164	0	0.0	0.077929	0.077968	0
[33]	*qnr (A,B,S)*	TyS	18	0	0.0	0.231477	0.232495	0
[34]	*qnr (A,B,S)*	TyS	89	0	0.0	0.105605	0.105703	0
[35]	*qnr (A,B,S)*	TyS	201	1	0.5	0.170087	0.070447	0.004975
[36]	*qnr (A,B,S)*	TyS	48	0	0.0	0.143348	0.143592	0
[36]‡	*qnr (A,B,S)*	TyS	16	0	0.0	0.244979	0.246183	0
[36]	*qnr (A,B,S)*	TyS	15	0	0.0	0.25268	0.254	0
[36]‡	*qnr (A,B,S)*	TyS	10	0	0.0	0.306277	0.308607	0
[38]	*qnr (A,B,S)*	TyS	128	1	0.8	0.212998	0.088216	0.007813
[63]	*qnr (A,B,S)*	TyS	11	0	0.0	0.292843	0.294884	0
[31]	Inv-salm	NTS	14110	840	6.0	0.493093	0.008418	0.059532
[32]	Inv-salm	NTS	9634	233	2.4	0.31262	0.010188	0.024185
[33]	Inv-salm	NTS	626	113	18.1	0.87895	0.039952	0.180511
[34]	Inv-salm	NTS	4674	196	4.2	0.412962	0.014626	0.041934
[36]	Inv-salm	NTS	1251	6	0.5	0.144125	0.028267	0.004796
[36]	Inv-salm	NTS	680	2	0.3	0.120641	0.038334	0.002941
[36]	Inv-salm	NTS	2476	1	0.0	0.048513	0.020095	0.000404

[36]	Inv-salm	NTS	1674	11	0.7	0.16587	0.024438	0.006571
[36]	Inv-salm	NTS	1021	7	0.7	0.171446	0.031288	0.006856
[36]	Inv-salm	NTS	1058	1	0.1	0.074206	0.030737	0.000945
[38]	Inv-salm	NTS	10133	94	0.9	0.193433	0.009934	0.009277
[31]	Inv-salm	TyS	14110	194	1.4	0.23535	0.008418	0.013749
[33]	Inv-salm	TyS	626	29	4.6	0.437278	0.039952	0.046326
[34]	Inv-salm	TyS	4674	89	1.9	0.277616	0.014626	0.019042
[35]	Inv-salm	TyS	9634	201	2.1	0.290246	0.010188	0.020864
[36]	Inv-salm	TyS	1251	54	4.3	0.420363	0.028267	0.043165
[36]	Inv-salm	TyS	680	9	1.3	0.236692	0.038334	0.013235
[36]	Inv-salm	TyS	2476	8	0.3	0.117176	0.020095	0.003231
[36]	Inv-salm	TyS	1674	15	0.9	0.192666	0.024438	0.008961
[36]	Inv-salm	TyS	1021	3	0.3	0.116808	0.031288	0.002938
[36]	Inv-salm	TyS	1058	9	0.9	0.189647	0.030737	0.008507
[38]	Inv-salm	TyS	10133	380	3.8	0.390007	0.009934	0.037501

Inv-salm, invasive salmonellosis; N, number of isolates; n, number positive; NTS, non-typhoidal *Salmonella;* P, proportion; Ref., reference; Se, standard error; t, double arcsine estimate, TyS, typhoidal *Salmonella*.
‡ Tanzania and Madagascar, and Senegal and Guinea-Bissau were combined.
